# Pathological mechanisms and research progress of multilevel intervention therapy for coronary heart disease

**DOI:** 10.3389/fimmu.2026.1816199

**Published:** 2026-04-13

**Authors:** Zhengyu Tu, Yuxin Wei, Yuanpeng Liao, Zhengdong Wan, Zhongquan Zhou, Jiawei Guo

**Affiliations:** 1 Department of Cardiology, The First Affiliated Hospital of Yangtze University, Jingzhou, China; 2 Department of Pharmacology, School of Medicine, Yangtze University, Jingzhou, China; 3 Department of Vascular and Endovascular Surgery, The First Affiliated Hospital of Yangtze University, Jingzhou, China

**Keywords:** atherosclerosis, coronary heart disease, endothelial dysfunction, metabolism-inflammation-immunity network, multi-level intervention therapy, vulnerable plaque

## Abstract

Coronary heart disease (CHD) is a chronic cardiovascular disease with coronary atherosclerosis as the main pathological basis. Its occurrence and progression are not a simple process of lipid deposition, but a systemic pathological process jointly driven by metabolic disorders, chronic low-grade inflammation, and immune imbalance. Recent studies have shown that endothelial dysfunction is a key event in the early occurrence of CHD. Abnormal lipid metabolism, immune cell infiltration, and continuous activation of inflammatory signals together promote plaque formation, maturation, and destabilization. Especially under the regulation of the metabolism-inflammation-immunity network, a positive feedback loop is formed between immune cell metabolic reprogramming, macrophage polarization, inflammasome activation, and oxidative stress response, which accelerates the progression of atherosclerosis and increases the risk of acute coronary events. Traditional therapeutic strategies centered on lipid-lowering, antiplatelet therapy, and revascularization can reduce the incidence of short-term events, but there are still limitations in long-term risk control and fundamental reversal of the disease. From a narrative perspective, this article summarizes the research progress of CHD in pathological mechanisms, metabolism and immune regulation, plaque instability, and risk factors from a systematic perspective and focuses on discussing the potential value of multilevel and systematic intervention strategies in the precise prevention and treatment of CHD, providing a theoretical basis for future translational research and personalized treatment.

## 1 Introduction

Coronary heart disease (CHD) is also known as coronary atherosclerotic heart disease (1). Atherosclerotic plaques form in the coronary arteries and continue to develop (2). This process leads to stenosis or even obstruction of the coronary lumen, resulting in insufficient blood supply and oxygen supply to the myocardium (3). As a type of chronic cardiovascular disease, it has long been regarded as a focal vascular disease, with the core characteristic of coronary lumen stenosis (4). Angina pectoris, myocardial infarction, heart failure, and even sudden death are all possible consequences (5).

However, in-depth basic and clinical research has continuously expanded people’s understanding of CHD (6). A large number of evidence have shown that the cause of CHD is not a simple process of passive lipid deposition in the vascular wall (7). It is a systemic and chronic disease. Multiple factors such as abnormal lipid metabolism, chronic mild inflammation, immune abnormalities, and endothelial dysfunction jointly promote its occurrence and development (8, 9). Coronary atherosclerosis is only the main manifestation of this complex pathological process in the coronary system, and its essence is systemic metabolic homeostasis imbalance (10). Currently, CHD is defined as a disease highly related to metabolism, inflammation, and immunity (11). Its occurrence, development, and final outcome are regulated by multiple levels such as molecules, cells, tissues, and systems (12).

There are clear criteria for the classification of CHD, which is mainly divided into two categories: stable CHD and ACS (acute coronary syndrome) based on clinical manifestations, pathological basis, and pathogenesis (13). Acute coronary syndrome also includes unstable angina pectoris, NSTEMI (non-ST-segment elevation myocardial infarction), and STEMI (ST-segment elevation myocardial infarction) (14). Stable angina pectoris is mostly caused by relatively stable atherosclerotic plaques. Patients have predictable myocardial ischemia symptoms during physical activity or emotional excitement. Its pathological root is mainly the fixed stenosis of the coronary lumen, leading to an imbalance between myocardial blood supply and demand (15).

In contrast, unstable angina pectoris and acute myocardial infarction are mostly caused by the rupture or erosion of vulnerable plaques (16). After plaque problems occur, platelet aggregation and thrombosis occur. Such diseases are characterized by sudden onset and rapid progression (17). Even within the same type of disease, the clinical manifestations of CHD patients are significantly different (18). Some patients have mild coronary stenosis but experience severe ischemia symptoms. In contrast, other patients have obvious coronary stenosis but no discomfort symptoms for a long time (19).

This phenomenon indicates that the clinical manifestations of CHD are not only determined by whether the coronary arteries are stenotic (20). Coronary microcirculatory function, endothelial reactivity, inflammatory status, and myocardial metabolic adaptability are all closely linked to clinical syndromes (21). In recent years, new concepts have emerged like non-obstructive coronary artery disease, coronary microvascular dysfunction, and INOCA (ischemia with no obstructive coronary artery disease) (22).

These concepts are challenging the old disease classification method. The old method focused on coronary stenosis degree and highlighted the key role of functional abnormalities and systemic regulatory imbalances in the disease (23). At the same time, the old method exposed the shortcomings of the traditional classification system. The traditional classification system, however, cannot explain complex clinical phenomena clearly (24).

Over the past few decades, in the diagnosis and treatment of CHD, researchers have made great progress, but the mortality rate of CHD remains high (25). This fact shows that the current CHD treatment system still has deficiencies in many sides.

In the diagnosis of early CHD, traditional imaging examinations often focus on existing harms. Those traditional examinations have limited ability to detect metabolic imbalances, inflammatory activation, and endothelial dysfunction, for these changes occur before plaque formation. As a result, high-risk people do not receive timely intervention sometimes; they miss the best treatment opportunity before obvious and severe symptoms appear.

In terms of CHD treatment, researchers still focus on lipid-lowering therapy, antiplatelet therapy, and revascularization. These methods can indeed significantly reduce the incidence rate in the short term, but their effect on maintaining long-term CHD stability is not ideal (26). Different patients respond differently to the same treatment plan. This means that there is no universal treatment model applicable to all patients; that is, personalized and precise treatment has great room for development.

In the long run, CHD is a typical chronic disease. Even if patients receive standardized treatment, they still face long-term cardiovascular risks. This indicates that treating only local lesions is difficult to cure CHD. It means that treating only regional lesions is not enough to completely cure CHD. We must understand the pathogenesis of CHD from a systemic level, then we can cure the disease once and for all.

One core challenge in current CHD research is the gap between basic research and clinical practice. Most research results show that inflammation, immunometabolism, and epigenetic regulation play key roles in CHD, but only a small part of these findings have been applied to clinical treatment.

At the basic research level of CHD, many cell types, signaling pathways, and regulatory networks are involved, results from different research models are still paradoxical, and animal models have their limitations. Moreover, the patient groups are highly diverse, and many patients are accompanied by metabolic diseases, which greatly increases the difficulty of understanding the research results.

In future CHD research, we need a comprehensive framework. Only by integrating the molecular mechanisms, systemic regulation, and clinical application of CHD can we effectively apply the findings of basic research to clinical practice (27).

This review will comprehensively sort out the research progress of CHD in pathogenesis, diagnostic technology, and treatment methods from a systemic perspective. Among them, this article focuses on the core role of the metabolism, inflammation, and immune network in the occurrence and development of CHD and clarifies the relationship between regulations at different levels. At the same time, it will discuss the possible development directions of future CHD treatment in combination with the latest clinical cases.

To ensure the comprehensiveness and rigor of this review, a systematic literature search was conducted following the PRISMA (Preferred Reporting Items for Systematic Reviews and Meta-Analyses) guidelines. The literature search covered five core databases (PubMed, Embase, Web of Science, CNKI, and WanFang) with a time frame from database establishment to October 2025. The key search terms included CHD, atherosclerosis, metabolism-inflammation-immunity network, vulnerable plaque, and multilevel intervention therapy (English and Chinese corresponding terms).

**Figure 1:**
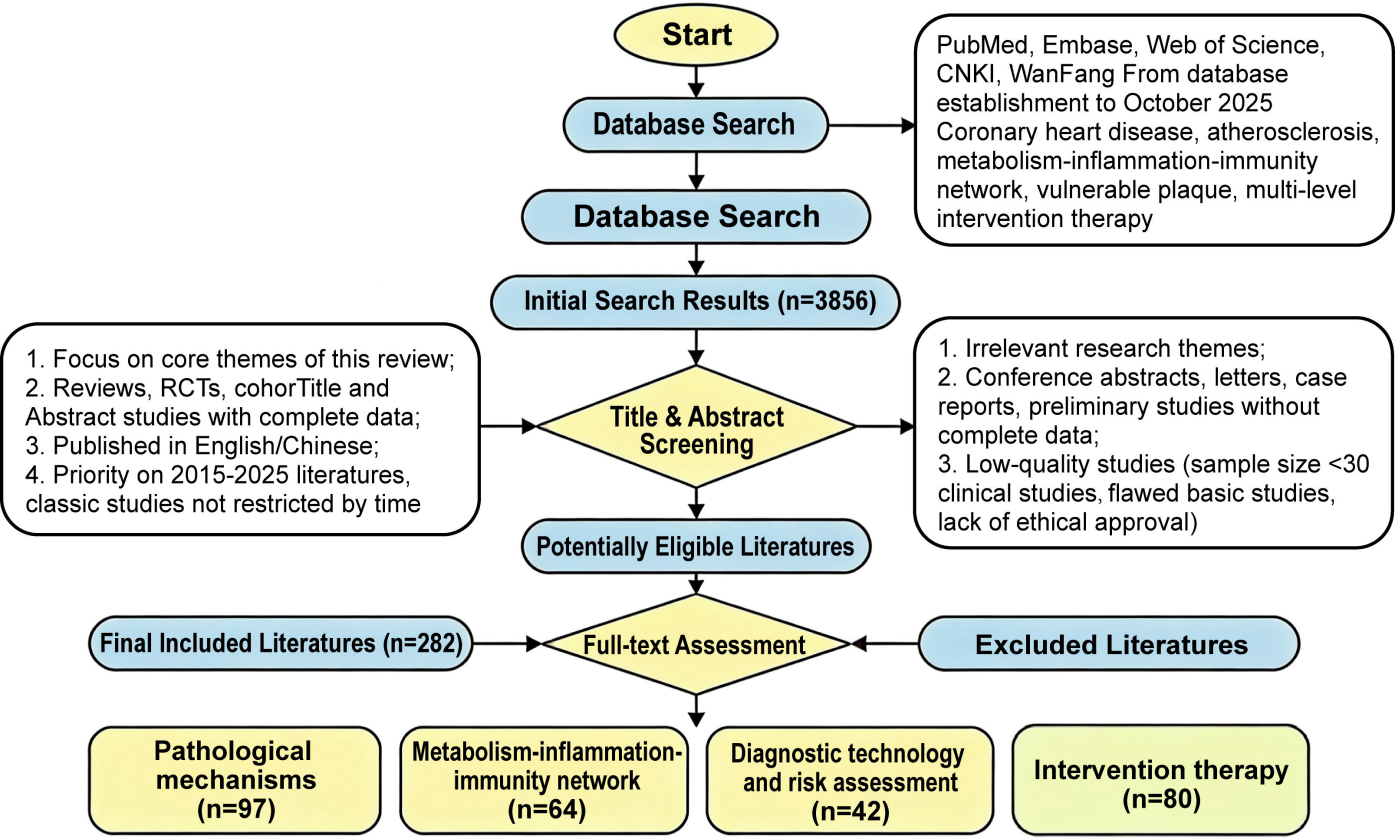
Flowchart of systematic literature search and study selection for this review. Letter *n* represents the number of articles included during a certain retrieval process.

A total of 3,856 literatures were initially retrieved, and 1,213 duplicate articles were excluded first. Subsequently, 2,031 literatures were excluded based on title and abstract screening (inconsistent with the review theme, non-full-text publications, or low-quality preliminary studies). Finally, 285 literatures were included after full-text evaluation, including 97 articles on pathological mechanisms, 64 on metabolism-inflammation-immunity network regulation, 42 on diagnostic technology and risk assessment, and 82 on intervention therapy. The inclusion criteria were as follows: (I) focusing on the core themes of this review; (II) being reviews, RCTs (randomized controlled trials), cohort studies, or basic experimental studies with complete data; (III) published in Chinese or English; (IV) priority given to literatures published in the past 10 years (2015–2025), whereas classic foundational studies were not restricted by publication time. The exclusion criteria were as follows: (I) irrelevant research themes; (II) conference abstracts, letters, case reports, or preliminary studies without complete data; (III) duplicate publications or non-Chinese/English literatures; (IV) low-quality studies (clinical studies with sample size <30, basic studies with flawed experimental design, or lack of ethical approval). The detailed process of literature search and selection is shown in the flowchart (**Figure 1**).

## 2 Pathological basis and pathogenesis of coronary heart disease

### 2.1 Headings

Coronary atherosclerosis is the pathological basis for the development and progression of CHD. It is not a static process but a long-term, dynamic development involving changes in vascular wall structure, disorders of lipid metabolism, chronic inflammatory responses, and complex interactions between various types of cells (28–30).

**Figure 2:**
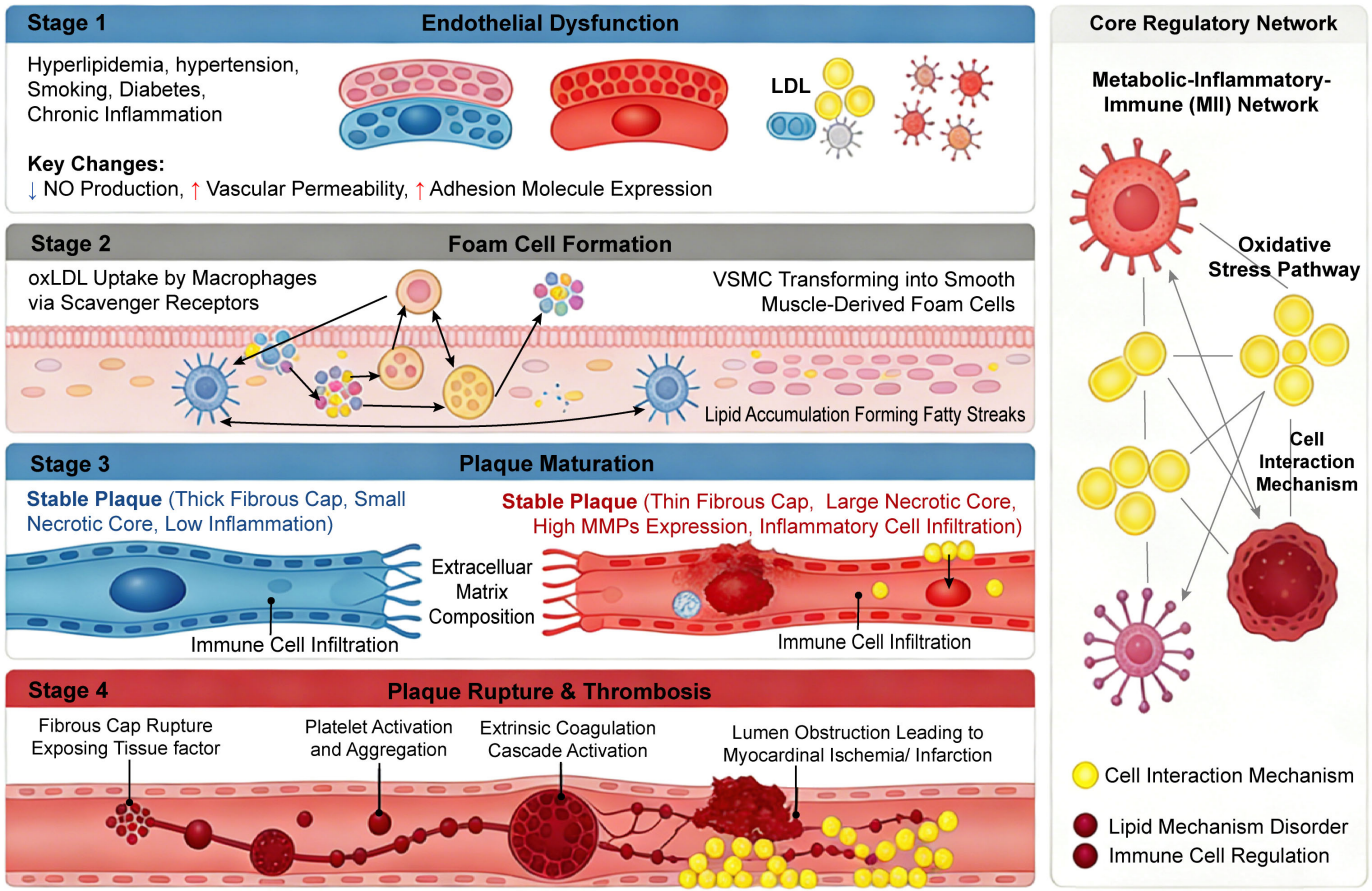
Pathological progression mechanism of coronary atherosclerosis from endothelial dysfunction to acute coronary syndrome: core molecular and cellular events. Abbreviations: CD36, cluster of differentiation 36; ICAM-1, intercellular adhesion molecule-1; LDL, low-density lipoprotein; MCP-1, monocyte chemoattractant protein-1; MMPs, matrix metalloproteinases; NO, nitric oxide; oxLDL, oxidized low-density lipoprotein; SR-A, scavenger receptor A; VCAM-1, vascular cell adhesion molecule-1; VSMC, vascular smooth muscle cell.

The development of coronary atherosclerosis does not proceed in a straight line but goes through a series of continuous stages. CHD develops step by step and starts with functional abnormalities, and then leads to structural damage of the vascular wall. The structural damage begins with early reversible changes and finally turns into irreversible lesions (31). To understand the pathogenesis of CHD, we need to know clearly about the whole process of coronary atherosclerosis. The whole process includes its occurrence, development, and final disease induction, as is shown in **Figure 2**.

#### 2.1.1 Initial stage of vascular endothelial injury

Vascular endothelial dysfunction is a well-recognized early sign of coronary atherosclerosis and serves as the initiating pathological event driving disease progression (32). Under normal physiological conditions, the vascular endothelium maintains vascular homeostasis by acting as a physical barrier and secreting bioactive factors such as nitric oxide and prostacyclin (33). However, prolonged exposure to pathogenic factors—including hyperlipidemia, hypertension, and diabetes—disrupts this stable state, leading to heterogeneous endothelial dysfunction phenotypes. These include EndMT (endothelial-to-mesenchymal transition), pro-inflammatory activation, and barrier dysfunction, which collectively promote the initiation and progression of atherosclerosis (34, 35).

EndMT is a key phenotypic switch where endothelial cells lose their morphology and tight junction proteins while acquiring mesenchymal markers and migratory capacities (36). Factors like oxidized low-density lipoprotein (oxLDL) and transforming growth factor-β (TGF-β) induce EndMT through pathways such as Smad2/3 (mothers against decapentaplegic homolog 2/3) and Wnt/β-catenin (wingless/integrated β-catenin) (37). These transdifferentiated cells contribute to plaque formation by secreting extracellular matrix proteins and migrating into the intima, accelerating intimal hyperplasia (38). Clinical studies confirm that EndMT markers are highly expressed in early lesions, and inhibiting this process reduces plaque burden (39).

The pro-inflammatory endothelial phenotype involves pattern recognition receptors activating NF-κB (nuclear factor-κB) and MAPK (mitogen-activated protein kinase) signaling, leading to upregulated adhesion molecules and chemokines (40). This inflammation exhibits memory through epigenetic changes, allowing sustained recruitment of immune cells to the vascular wall even after initial stimuli are removed (41). Closely related is endothelial barrier dysfunction, where oxidative stress and pro-inflammatory cytokines impair intercellular junctions, increasing vascular permeability and facilitating lipid and monocyte infiltration (42).

These dysfunctional phenotypes are governed by intrinsic mechanisms including oxidative stress imbalance, metabolic reprogramming toward glycolysis, and epigenetic regulation (43, 44). The consequences are amplified through interactions with other cells. Dysfunctional endothelial cells recruit monocytes that differentiate into macrophages, further amplifying inflammation (45), and they also induce vascular smooth muscle cell proliferation while receiving signals that aggravate barrier dysfunction. In addition, they promote platelet adhesion, creating a pro-thrombotic microenvironment (46).

**Figure 3:**
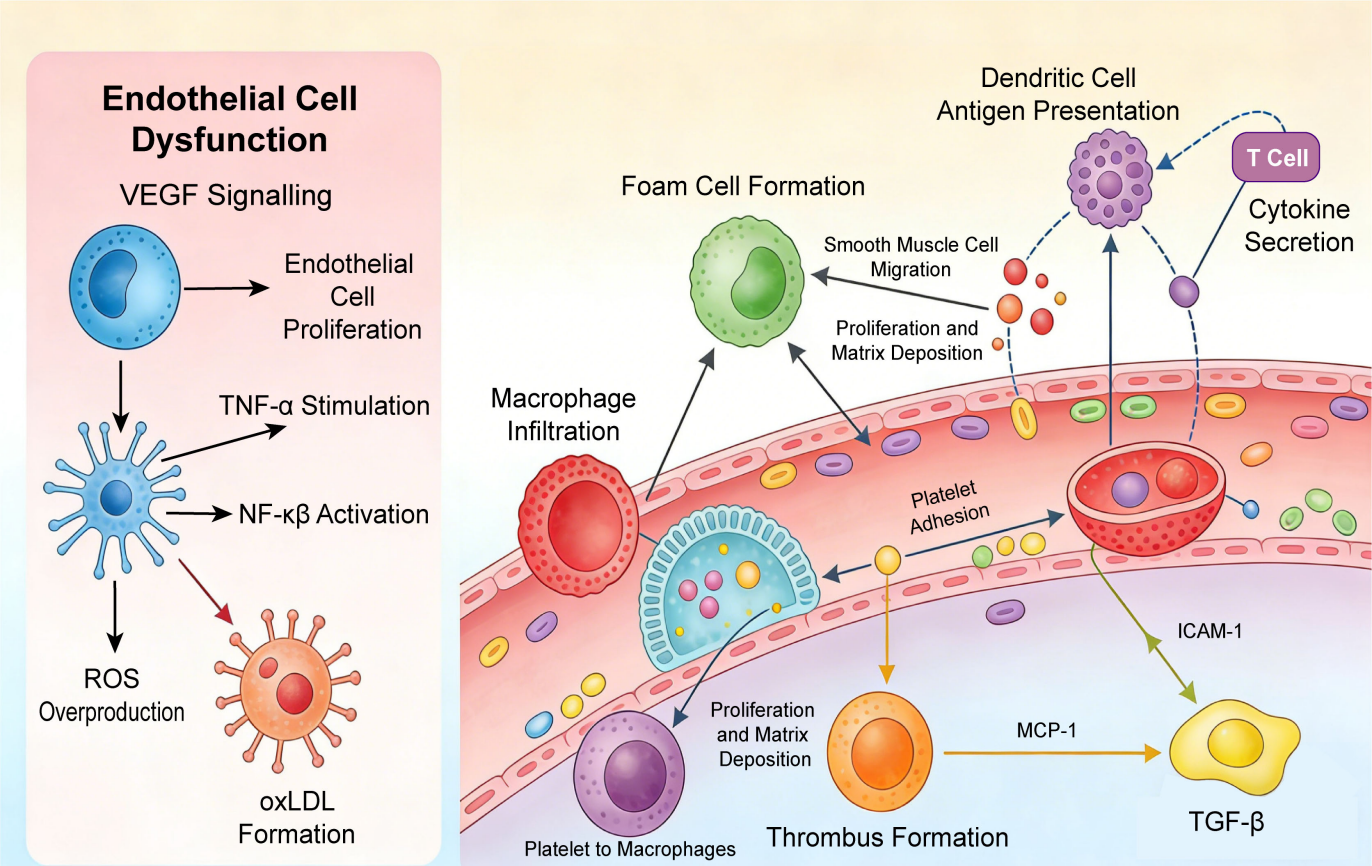
Schematic diagram of endothelial dysfunction-mediated atherosclerotic initiation and cellular crosstalk. Abbreviations: ICAM-1, intercellular adhesion molecule-1; IL-1β, interleukin-1β; NF-κB, nuclear factor-κB; oxLDL, oxidized low-density lipoprotein; TNF-α, tumor necrosis factor-α; VCAM-1, vascular cell adhesion molecule-1; VEGF, vascular endothelial growth factor.

Ultimately, endothelial dysfunction initiates atherosclerosis by enabling lipid infiltration, recruiting inflammatory cells, promoting vascular remodeling, and increasing plaque vulnerability (47). Notably, although undetectable by conventional imaging, this represents a reversible stage of atherosclerosis. Targeting specific phenotypes, through eNOS (endothelial nitric oxide synthase) activators or anti-inflammatory therapies, may delay or reverse early lesions, highlighting its potential as a therapeutic target for primary prevention of CHD (48). The schematic diagram of endothelial dysfunction-mediated atherosclerotic initiation and cellular crosstalk is shown in **Figure 3**.

#### 2.1.2 Lipid deposition and foam cell formation

After the increase in endothelial permeability, circulating LDL (low-density lipoprotein) particles take the opportunity to enter the vascular intima and stay locally. These retained LDL will eventually form oxLDL under the combined action of oxidative stress and inflammatory responses (49). OxLDL has strong pro-inflammatory effects and immunogenicity, playing a key role in exacerbating atherosclerosis (50).

OxLDL activates vascular wall cells through various pathways. Monocytes migrate to the intima under the guidance of chemokines [such as MCP-1 (monocyte chemoattractant protein-1)] and differentiate into macrophages. These macrophages extensively take up oxLDL through scavenger receptors [such as SR-A (scavenger receptor A) and CD36 (cluster of differentiation 36)], but this uptake process is not regulated by negative feedback of intracellular cholesterol content. Cholesterol esters accumulate continuously in the cells, eventually forming foam cells (51).

The appearance of foam cells indicates the formation of fatty streaks, which is the earliest morphological manifestation of atherosclerosis. However, foam cells are not just storage warehouses for lipids; they are also highly active inflammatory cells that secrete various pro-inflammatory cytokines, chemokines, and matrix-degrading enzymes, further recruiting more immune cells and amplifying local inflammatory responses, forming a positive feedback that promotes lipid deposition and inflammatory activation, worsening the condition.

A major important finding in recent studies is that vascular smooth muscle cells also participate in the formation of foam cells. Under specific stimuli, the function of vascular smooth muscle cells changes, acquiring phagocytic ability similar to macrophages, participating in lipid accumulation and plaque construction, making the cellular origin of coronary atherosclerosis more complex (52).

#### 2.1.3 Maturation and stability of atherosclerotic plaques

With the continuous deposition of lipids and the progression of inflammatory responses, coronary atherosclerosis gradually develops from fatty streaks to mature plaques with a more complex structure. A mature atherosclerotic plaque usually consists of three parts: a lipid-rich necrotic core, a fibrous cap covering the surface, and surrounding inflammatory cells and connective tissue (53).

Vascular smooth muscle cells migrate from the media to the intima during plaque maturation. After reaching the intima, vascular smooth muscle cells secrete collagen and other extracellular matrix components to jointly form the fibrous cap structure. The thickness and composition of the fibrous cap are the key determinants of plaque stability. Plaques with a thicker fibrous cap and fewer inflammatory cells are usually more stable and often lead to chronic vascular wall stenosis; vulnerable plaques with a thin fibrous cap and more inflammatory cells have a higher risk of rupture (54).

The stability of atherosclerotic plaques is regulated by various factors. Matrix metalloproteinases secreted by inflammatory cells degrade the collagen fibers in the fibrous cap, making the structure of the fibrous cap more fragile. Continuous lipid accumulation and cell apoptosis also continuously expand the volume of the necrotic core, increasing the pressure on the plaque; changes in local blood flow also affect the distribution and rupture risk of the plaque.

Once an atherosclerotic plaque ruptures or is eroded, the consequences are very serious. The exposed lipid core and tissue factors will quickly activate platelets, triggering a coagulation cascade reaction and forming a thrombus, directly triggering acute coronary syndrome. This process has no necessary connection with the size of the plaque but depends more on the inherent characteristics and stability of the plaque. Some lesions with only moderate stenosis can also cause severe CHD. The key pathological stages and core mechanisms in the occurrence and development of CHD are shown in **Table 1**.

**Table 1:** Key pathological stages and core mechanisms of CHD development and progression.

Pathological stage	Key cell types	Main signaling axes/molecules	Histopathological characteristics	Plaque instability	Clinical relevance	References
Endothelial dysfunction	Endothelial cells	Adhesion axis (VCAM-1↑, ICAM-1↑); vasomotor axis (eNOS↓, NO↓)	Vascular permeability increased; endothelial cells transition from anti-inflammatory/anticoagulant to pro-inflammatory/pro-coagulant phenotype	None	Early silent stage of CHD; pre-plaque lesion	Gimbrone MA Jr, 2016 (Circ Res)(10)
Lipid deposition and foam cell formation	Macrophages, VSMC	Lipid metabolism axis (oxLDL, CD36, SR-A); inflammation axis (MCP-1↑)	Fatty streak formation; massive cholesterol ester accumulation in foam cells; local inflammation amplification	Low	Initiation of atherosclerotic plaque	Tabas I, 2007 (Circulation)(8)
Mature plaque formation	VSMC, immune cells (macrophages, T cells)	Extracellular matrix axis (collagen synthesis); protease axis (MMPs↓)	Structured plaque with lipid-rich necrotic core and thick fibrous cap; balanced inflammation and repair	Very low	Stable CHD; chronic vascular stenosis	Virmani R, 2006 (J Am Coll Cardiol)(7)
Vulnerable plaque stage	Macrophages, T cells (Th1, Th17)	Inflammation axis (IL-1β↑, TNF-α↑, NLRP3 inflammasome activation); protease axis (MMPs↑)	Thin fibrous cap; expanded necrotic core; massive inflammatory cell infiltration; collagen fiber degradation	Very high	High risk of ACS; plaque rupture/thrombosis	Naghavi M, 2003 (Circulation)(55)

Acute coronary syndrome; CHD, coronary heart disease; eNOS, endothelial nitric oxide synthase; ICAM-1, intercellular adhesion molecule-1; IL-1β, interleukin-1β; MCP-1, monocyte chemoattractant protein-1; MMPs, matrix metalloproteinases; NLRP3, NOD-like receptor family pyrin domain containing 3; NO, nitric oxide; oxLDL, oxidized low-density lipoprotein; SR-A, scavenger receptor A; TNF-α, tumor necrosis factor-α; VCAM-1, vascular cell adhesion molecule-1.

### 2.2 Vascular endothelial dysfunction and vascular homeostasis imbalance

Vascular endothelial dysfunction connects pathogenic factors with coronary atherosclerosis, playing a fundamental and amplifying role in the occurrence, development, and pathogenic process of CHD. Under normal conditions, the body’s vascular homeostasis relies on the precise regulation of vascular tone, inflammatory response, balance between coagulation and fibrinolysis, and metabolism of vascular wall cells by endothelial cells. Once the function of endothelial cells is impaired and this balance is broken, CHD will continue to develop.

#### 2.2.1 Endothelial cell injury and functional changes of endothelial cells

Endothelial cell injury is not simply cell death or structural destruction, but a dynamic pathological state of functional abnormalities. Under the long-term action of CHD-related pathogenic factors, endothelial cells undergo a series of functional changes, the most representative of which are decreased vasomotor regulation ability and loss of anti-inflammatory and anticoagulant properties (56).

A key change in the occurrence of endothelial dysfunction is the decrease in the activity of eNOS, leading to reduced bioavailability of NO (nitric oxide) (57). Enhanced oxidative stress, uncoupling of endothelial nitric oxide synthase, and lack of substrates and cofactors all result in insufficient NO production, weakening the vasodilator response, promoting platelet aggregation, relatively increasing the level of endothelin-1 produced by endothelial cells, further enhancing vascular contractility, and causing imbalance in vasomotor function (58).

In terms of inflammatory regulation, damaged endothelial cells undergo obvious transformation. Instead of inhibiting the adhesion of inflammatory cells, they become pro-inflammatory, with increased expression of various adhesion molecules and chemokines, making it easier for monocytes and T lymphocytes to adhere to the vascular wall and migrate into it. This pro-inflammatory state not only promotes early lesions of atherosclerosis but also maintains a chronic inflammatory microenvironment in mature plaques (59, 60).

When the endothelial barrier function is damaged, vascular permeability increases, and lipids, inflammatory mediators and coagulation factors can enter the vascular wall more easily (61). These substances worsen the condition in various ways, all these changes can cause endothelial dysfunction, and the dysfunction is a key cause of CHD.

#### 2.2.2 Relationship between vasoconstriction and thrombosis

Abnormal vasoconstriction and thrombosis directly lead to CHD, and both are closely related to endothelial dysfunction. Under normal physiological conditions, endothelial cells release factors like NO and prostacyclin; these factors inhibit platelet activation and prevent vascular smooth muscle contraction, thus keeping blood flow smooth (62).

When there is endothelial dysfunction, the inhibiting effect will be greatly reduced, and blood vessels are more likely to have spasmodic contraction, especially in areas with coronary stenosis or more plaques. Changes in local blood flow will not only worsen myocardial ischemia but also increase blood flow pressure and make plaques more unstable and raises the risk of lesions (55, 63).

At the same time, the anticoagulant function of endothelial cells will gradually disappear. The expression of tissue factor upregulates, and plasmin activity reduces. Platelet adhesion ability is enhanced, and it promotes thrombosis (64, 65). Once a plaque ruptures or is eroded, the exposed matrix components and tissue factors quickly activate the coagulation cascade; it is when a thrombus forms and blocks coronary blood flow in a short time, and it eventually leads to unstable angina or acute myocardial infarction (66).

Vasoconstriction and thrombosis are not separate; they reinforce each other and form a vicious circle. Vasospasm worsens local ischemia and damages the endothelium further, and thrombosis changes blood flow status, making vasoconstriction more serious. This principle can also reasonably explain why some patients still have severe CHD even with low coronary artery stenosis.

#### 2.2.3 Interactive effects between endothelial cells and smooth muscle cells

The dynamic coordination between vascular endothelial cells and vascular smooth muscle cells is the key to maintaining vascular homeostasis. This homeostasis includes both the stability of vascular structure and the normalcy of function. In healthy blood vessels, endothelial cells function through paracrine. It can regulate the contraction, proliferation, and migration of smooth muscle cells, thereby maintaining the integrity and elasticity of the vascular wall structure (67).

However, under the pathological state related to CHD, the signal communication between these cells changes. Damaged endothelial cells secrete more growth factors, inflammatory mediators, and contractile factors. These substances, can promote the transformation and the proliferative capacity of smooth muscle cells and migrate to the intima. As a result, these substance participate in the deposition of extracellular matrix and promote the formation of plaque structure (68, 69).

Smooth muscle cells, in turn, also affect endothelial cells. They secrete inflammatory factors and chemotactic signals; as a result, these cells aggravate the inflammatory state of the endothelium and further damage endothelial function. The two-way interactive effect forms a positive feedback loop and continuously plays a role in the progression of atherosclerosis and vascular remodeling (70).

In recent years, new studies have found that under specific conditions, smooth muscle cells can acquire some functional characteristics. These functions are similar to that of immune cells, and they can also phagocytose lipids and express inflammation-related molecules, just like immune cells (71). These studies have expanded our new understandings about the mechanism of vascular homeostasis imbalance. In addition, they provide a new perspective for us to understand the complexity of CHD.

### 2.3 Relationship between abnormal lipid metabolism and coronary heart disease

Abnormal lipid metabolism is a core driving factor for CHD pathogenesis. The role of abnormal lipid metabolism runs through the entire process of atherosclerosis. It involves changes in blood lipid composition and abnormal function of lipid particles, and at the cellular level, it also includes disorders of lipid processing function. These abnormalities change the state of lipid deposition in the coronary intima, activate inflammatory responses, and also affect the stability of plaques (72, 73). Recent studies have found that abnormal lipid metabolism not only is the initiating factor of CHD but also can regulate pathways related to immunity, inflammation, and vascular remodeling, further increasing the incidence of CHD (74, 75).

#### 2.3.1 Lipid basis of atherosclerosis

Low-density lipoprotein (LDL) is a key lipid carrier for the formation of atherosclerosis, and its concentration and oxidation state are closely related to the risk of CHD, usually positively correlated. When vascular endothelial permeability increases, LDL particles in the blood circulation enter the intima, stay locally, and undergo oxidation, eventually becoming oxLDL with high inflammatory activity (76, 77). These oxLDL are taken up by macrophages, prompting macrophages to form foam cells (78). At the same time, it can activate endothelial cells and vascular smooth muscle cells, induce the expression of pro-inflammatory factors and matrix-degrading enzymes, and further damage vascular homeostasis.

Unlike LDL, HDL (high-density lipoprotein) has a protective effect on CHD. It participates in the reverse cholesterol transport process, transporting cholesterol from the intima to the liver, where these cholesterols are metabolized and excreted from the body, reducing lipid accumulation in blood vessels. Moreover, HDL can also exert antioxidant and anti-inflammatory effects, improve endothelial function, slow down plaque formation, and reduce plaque instability. However, if the function of HDL is impaired or its quantity is insufficient, this protective mechanism will be weakened, and the risk of atherosclerosis will also increase significantly (79, 80).

The interaction between LDL and HDL is an important link in the balance of blood lipid metabolism. Excess LDL or dysfunction of HDL will break this balance, triggering a series of pro-inflammatory and pro-coagulant reactions, creating conditions for the early formation of CHD.

#### 2.3.2 Correlation between triglycerides and coronary heart disease

TG (triglyceride) is another important type of lipid in plasma. It is closely related to the risk of CHD and is an undeniable risk factor (81). Hypertriglyceridemia can increase the possibility of atherosclerosis in the human body through various mechanisms (82). Under the state of hypertriglyceridemia, it is usually accompanied by the formation of sdLDL (small, dense low-density lipoprotein). This type of LDL particle is more likely to penetrate the endothelium and is also prone to oxidation, which is then taken up by related cells to form foam cells. At the same time, lipoprotein particles rich in triglycerides will increase blood viscosity. Slow blood flow will promote plaque formation and aggravate vascular inflammation (83).

The state of hypertriglyceridemia also affects the function of high-density lipoprotein, leading to its dysfunction, weakening the efficiency of the reverse cholesterol transport process, and slowing down the clearance of cholesterol in the intima. Moreover, triglycerides can induce low-grade inflammation, damage endothelial function, and indirectly accelerate the progression of atherosclerosis. The results of clinical studies show that although LDL is still the main risk factor for CHD, patients with high triglyceride levels still have a significantly increased risk of CHD even if LDL is effectively controlled. This result also suggests that triglycerides are an important pathogenic factor of CHD, and this result has important clinical significance (84).

#### 2.3.3 Role of fatty acid metabolism in coronary heart disease

Fatty acids are important substrates for cellular energy metabolism, and their metabolic abnormalities also occupy an important position in the pathogenesis of CHD (85). Myocardial cells and vascular cells mainly rely on long-chain fatty acid oxidation to generate ATP to provide energy for their own activities (86). However, in the early stage of CHD, the metabolic balance between fatty acid oxidation and glycolysis is disrupted, leading to insufficient energy supply, impaired mitochondrial function, and massive production of ROS (reactive oxygen species). These changes all cause damage to cells and promote the development of CHD (87).

If fatty acids accumulate excessively or their metabolism is unbalanced, they can directly trigger inflammatory responses. Saturated fatty acids can activate TLR (Toll-like receptors) of macrophages and induce the activation of the NF-κB signaling pathway, thereby increasing the secretion of pro-inflammatory cytokines and aggravating inflammatory responses, whereas unsaturated fatty acids, especially ω-3 polyunsaturated fatty acids, have completely different effects. They have anti-inflammatory effects, and they can also improve endothelial function, help stabilize plaques, and play a protective role in blood vessels. This indicates that different types of fatty acids and metabolic states play different roles in the development of CHD (88).

Abnormal fatty acid metabolism also affects the phenotypic transformation of vascular smooth muscle cells and interferes with the signal communication between endothelial cells and smooth muscle cells, thereby promoting the formation of fibrous caps and leading to the structural remodeling of plaques. More and more studies have shown that fatty acid metabolism is closely linked to the immunometabolic network, and fatty acid metabolism has become an important regulatory node for the initiation and development of CHD and the formation of vulnerable plaques. This research result has a key impact on the development trajectory of CHD.

### 2.4 Role of inflammatory responses and immune cells in plaque formation

Atherosclerosis is no longer simply defined as a lipid deposition disease but is now generally recognized in the academic community as a chronic low-grade inflammatory disease (89). In the entire pathological process of CHD, the role of inflammatory responses is crucial. It not only participates in the formation of early plaques but also directly determines the stability of mature plaques and whether the plaques will rupture. Immune cells and inflammatory mediators construct a complex regulatory network inside and outside the vascular wall, which directly act on endothelial cells, smooth muscle cells, and foam cells, thereby affecting the construction and evolution of plaques (90, 91).

#### 2.4.1 Role of immune cells in plaque formation

Macrophages are the main type of immune cells in atherosclerotic plaques and the core executor of inflammation-driven process (92). Circulating monocytes migrate to the intima and differentiate into macrophages under the guidance of vascular endothelial adhesion and chemokines (93). These macrophages take up oxLDL to form foam cells and can secrete various pro-inflammatory cytokines, MMPs, and oxidative stress molecules, which not only participate in the formation of the plaque core but also directly damage the fibrous cap structure, increasing the vulnerability of the plaque (94).

T lymphocytes play a regulatory role in atherosclerosis (95). CD4^+^T cells can differentiate into multiple subtypes, including Th1, Th2, Th17, and Treg (regulatory T cells). Among them, Th1 cells secrete IFN-γ (Interferon-γ), activate macrophages and vascular smooth muscle cells, aggravate inflammatory responses, and promote the degradation of fibrous caps; Th2 and Treg cells secrete inhibitory factors such as IL-10 (interleukin-10) and triglyceride F-β, which have a protective effect on plaque stability and can reduce the risk of plaque rupture; Th17 cells and their secreted IL-17 (interleukin-17) are closely related to local inflammation amplification and smooth muscle cell apoptosis, which also greatly increases the vulnerability of the plaque (96). The proportion and functional state of T-cell subsets directly affect the characteristics of the plaque and determine the risk of CHD.

In addition to macrophages and T lymphocytes, NK cells (natural killer), dendritic cells, and B cells also participate in the pathogenic mechanism of CHD. They participate in local immune responses and regulate through antibody mediation (97). Although the specific mechanism of action of these cells has not been fully studied, there is evidence that they can indirectly affect the formation and progression of plaques by regulating the activity of macrophages and the level of inflammatory mediators.

#### 2.4.2 Relationship between inflammatory factors and the progression of coronary heart disease

As the main immune cells in atherosclerotic plaques, macrophages are the core executors of inflammation-driven atherosclerosis. Circulating monocytes adhere to the vascular endothelium and migrate to the intima under the guidance of chemokines. Monocytes reaching the intima differentiate into macrophages (98). Macrophages take up oxLDL to form foam cells and at the same time secrete various pro-inflammatory cytokines, MMPs, and oxidative stress molecules. These substances participate in the formation of the plaque core and directly damage the fibrous cap structure, making the plaque more prone to rupture (99).

In the pathological cascade driven by inflammatory factors, the CD40-CD40L signaling pathway plays a key regulatory role. This pathway not only is involved in T-cell and B-cell-mediated adaptive immune responses but also exists on the surface of macrophages, endothelial cells, and vascular smooth muscle cells. When CD40 binds to its ligand CD40L (CD40 ligand), it triggers downstream cascade reactions and promotes the secretion of pro-inflammatory cytokines, the release of matrix-degrading proteases, and the expression of adhesion molecules (100). This exacerbates local inflammatory infiltration and damages the structural stability of the fibrous cap.

Through animal experiments, researchers have confirmed that knocking out CD40L gene works, and blocking the pathway with neutralizing antibodies also works. Both methods can reduce the volume of atherosclerotic lesions, decrease the degree of plaque inflammation, and increase the fibrous component. In clinical studies, researchers have also found something important (101). The activation level of the CD40-CD40L pathway in patients with CHD is closely connected with plaque vulnerability, and also the risk of cardiovascular events.

In addition, this pathway can also induce endothelial cells and macrophages to express tissue factor, enhance the thrombogenic tendency of the plaque, and at the same time regulate the proliferation and migration of vascular smooth muscle cells, participating in the formation of fibrous plaques and vascular remodeling. As a core hub connecting inflammatory responses, immune activation, and plaque pathological remodeling, it provides an important molecular target for the targeted treatment of CHD (102).

### 2.5 Plaque rupture, thrombosis, and acute coronary heart disease

AMI (acute myocardial infarction), unstable angina pectoris, etc., are the final results of the development of coronary atherosclerosis, that is, CHD. The occurrence of these different types of CHD is mainly due to thrombosis formed after the rupture or erosion of mature plaques. Plaque rupture is not only a direct manifestation of local vascular structural imbalance but also the final result of long-term interaction between immune, inflammatory, and metabolic abnormalities (103). Understanding the mechanism of plaque rupture and thrombosis is the core to preventing acute CHD and guiding clinical intervention.

The inflammatory mechanism of atherosclerosis can be independently verified by allograft vasculopathy. As a special type of immune-mediated arteriosclerosis, this disease can occur rapidly without traditional atherosclerosis risk factors such as hyperlipidemia and hypertension. Its core mechanism is the immune response against the allogeneic class II histocompatibility antigen of donor cells, which activates the chronic cytokine-mediated immune response, thereby inducing concentric fibromatous arteriosclerosis. Studies have confirmed that coronary endothelial cells of cardiac allografts express class II histocompatibility antigens, and IFN-γ secreted by activated T cells is a key initiator of this pathological cascade reaction. IFN-γ deficiency can prevent the occurrence of coronary arteriosclerosis in mouse cardiac allografts without affecting myocardial rejection. This research result directly confirms that the inflammatory mechanism itself can drive the occurrence and development of arteriosclerosis, supplements the deficiencies of the traditional lipid deposition theory, and also provides independent pathological evidence support for the inflammatory intervention of atherosclerosis. At the same time, it confirms the synergistic effect of innate and adaptive immunity in vascular lesions, which is mutually corroborated with the inflammatory regulatory network of atherosclerosis (104).

#### 2.5.1 Mechanism and clinical manifestations of plaque rupture

Plaque rupture usually occurs on vulnerable plaques, which are characterized by a thin fibrous cap and highly activated inflammatory cells (105). The mechanism of plaque rupture can be understood from several related aspects. The fibrous cap of the plaque is composed of smooth muscle cells and collagen fibers. MMPs secreted by inflammatory cells degrade these collagens and elastic fibers, reducing the strength of the fibrous cap and increasing the risk of rupture; at the same time, the apoptosis of smooth muscle cells and the decreased ability to synthesize collagen will further reduce the structural strength of the fibrous cap, which is a core problem in plaque rupture (106).

The core part of the plaque is also rich in lipids, cholesterol crystals, and apoptotic cells. The continuous accumulation of these substances expands the volume of the plaque core, thereby forming a high-stress area. The stress difference between the lipid core and the fibrous cap makes the fibrous cap more prone to rupture under force. Therefore, the expansion and necrosis of the lipid core are important factors promoting plaque rupture in terms of structure (107).

Macrophages and T cells are continuously activated during plaque rupture. They produce a large number of pro-inflammatory cytokines such as IL-1β (interleukin-1β) and TNF-α (tumor necrosis factor-α), as well as ROS, which further damage the fibrous cap and also induce local cell apoptosis and tissue remodeling. The increase in inflammation level is closely related to plaque rupture (108).

In addition, abnormal local blood flow shear stress also has an impact (109). At the branching parts of blood vessels, endothelial dysfunction leads to stress concentration on the fibrous cap, which increases the probability of plaque rupture, triggers platelet adhesion, and exacerbates local inflammatory responses, forming a cycle of mutual promotion of rupture and thrombosis. Hemodynamic factors play a key regulatory role in this process.

Clinically, plaque rupture is often manifested as an acute coronary syndrome, including sudden angina pectoris, ST-segment elevation, and non-ST-segment elevation myocardial infarction. Some patients may even present with sudden death, which is particularly common when plaque rupture is accompanied by complete thrombosis (110).

#### 2.5.2 Thrombosis formation and the occurrence of acute myocardial infarction

When a plaque ruptures or is eroded, the lipid core and tissue factors under the vascular intima are exposed, rapidly initiating the thrombosis process (111). Platelets adhere to the injured site through collagen and glycoprotein VI, glycoprotein Ib–IX–V complex, and undergo aggregation and release reactions under the action of activating factors; at the same time, tissue factors trigger the extrinsic coagulation cascade reaction, leading to fibrin formation and rapid thrombus expansion (112, 113).

Thrombosis can partially or completely block coronary blood flow, leading to local myocardial ischemia and necrosis, triggering acute myocardial infarction (114). Clinically, complete thrombotic obstruction usually manifests as ST-segment elevation myocardial infarction (STEMI), whereas partial obstruction and minor plaque rupture mostly manifest as NSTEMI (non-ST-segment elevation myocardial infarction) or unstable angina pectoris (115).

It should be emphasized that thrombosis formation is not only affected by plaque structure but also regulated by systemic inflammation, hypercoagulable state of blood, platelet reactivity, and microvascular function (116). At most times, thrombosis and plaque rupture always happen together, and they can trigger acute CHD. The mechanism about thrombosis and plaque rupture relies heavily on the long-term cumulative effects of lipid deposition, immune inflammation, and endothelial dysfunction.

Recent studies have shown that antiplatelet, anticoagulant, and anti-inflammatory treatments can cut down the incidence of acute CHD significantly. It also proves indirectly that thrombosis plays a key role in causing CHD. Thus, identifying high-risk vulnerable plaques and thrombosis in patients is an important way to prevent and treat acute myocardial infarction (117).

## 3 Regulatory mechanisms of the metabolism-inflammation-immunity network

Recent research has shown that CHD is not just a local vascular disease but also a systemic disorder. CHD is driven by the close interaction of metabolism, inflammation, and immunity (118). These three systems do not work alone; they form a complex network, and in the network, changes in one system quickly affect the other two systems. The mutual regulation runs through the whole process of atherosclerosis, from initial endothelial injury to the final plaque rupture (119).

The MII (metabolism-inflammation-immunity) network is the core internal mechanism; it determines the occurrence and development of CHD and also explains why metabolic diseases like diabetes and obesity often lead to a higher risk of cardiovascular diseases (120). Studying the regulatory mechanisms of this network helps researchers to find new targets for the early intervention and precise treatment of CHD.

### 3.1 Immune cell metabolic reprogramming and atherosclerosis

**Figure 4:**
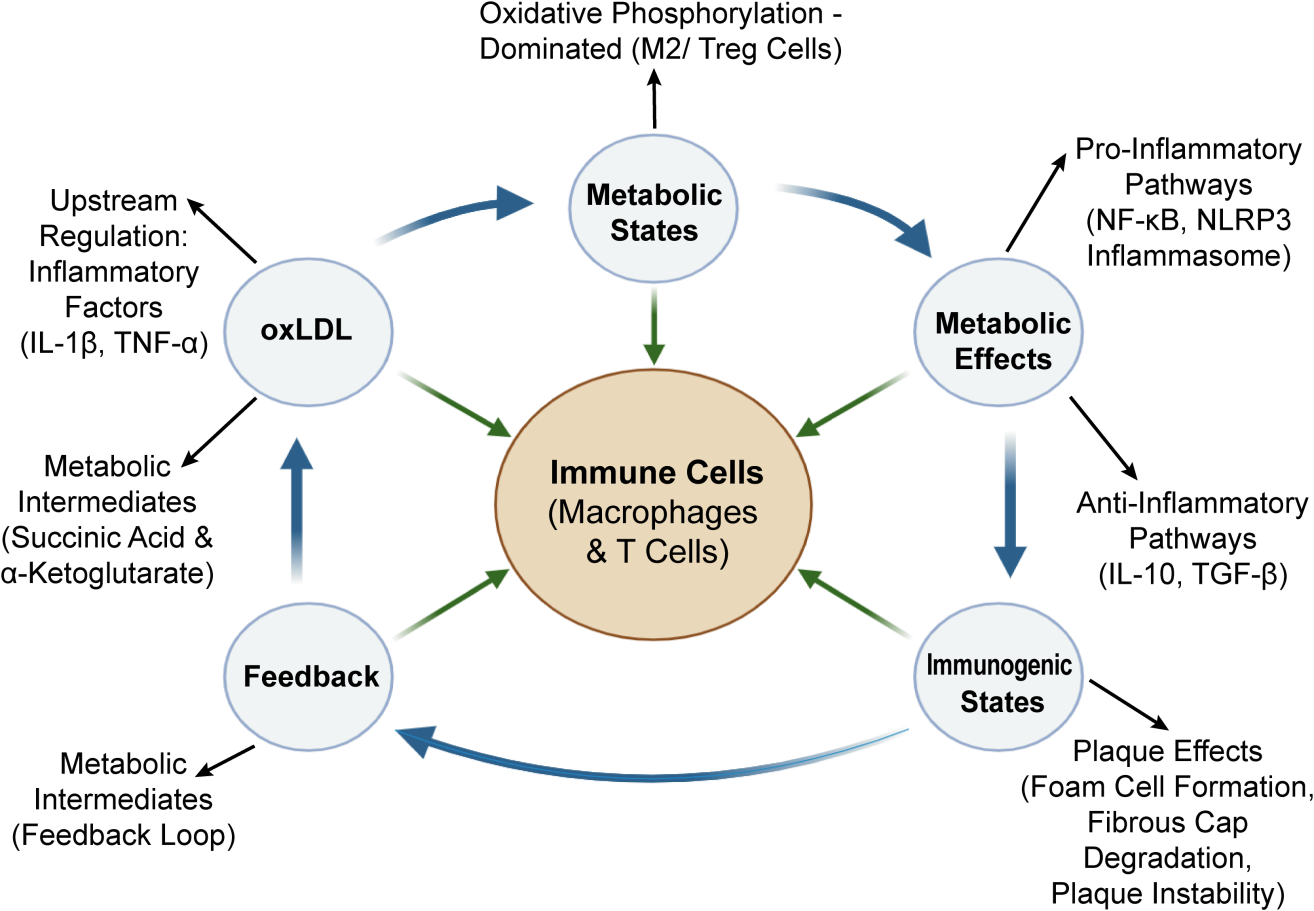
Core regulatory mechanism diagram of metabolic-inflammatory-immune (MII) network.

Pathological conditions, such as atherosclerosis, change immune cells. Immune cells alter their original metabolic pathways to adapt to the local microenvironment, and the change is called metabolic reprogramming (121). The reprogramming is not a passive reaction but an active regulatory process and directly determines the function and survival state of immune cells; after that, it affects the progression of the disease (122). Monocyte-macrophages, T cells, and dendritic cells all exhibit metabolic reprogramming, as is shown in **Figure 4**.

#### 3.1.1 Metabolic characteristics of macrophages in atherosclerotic plaques

Macrophages are the most important immune cells in plaques, and their metabolic state is closely linked to their polarization direction (123). In the early stage of inflammation, macrophages are mainly polarized to the M1 type and are in a pro-inflammatory state. They mainly rely on glycolysis to get energy, even when oxygen is sufficient (124). The metabolic pattern produces ATP quickly and also provides raw materials for the synthesis of pro-inflammatory factors. Therefore, it helps the inflammatory response amplify rapidly (125).

M2-type macrophages, in contrast, are in an anti-inflammatory state and mainly rely on oxidative phosphorylation to get energy (126). The stable metabolic pattern is good for tissue repair and inflammation resolution. In atherosclerotic plaques, the balance between M1 and M2 macrophages is often broken. At many times, there are more M1-type macrophages, and this leads to the persistence of local inflammation; it also causes the continuous destruction of plaque structure (127).

Studies have found that oxLDL can induce metabolic reprogramming of macrophages (128). It inhibits the activity of mitochondrial enzymes and forces macrophages to switch to glycolysis. The change not only promotes the formation of foam cells but also enhances the secretion of pro-inflammatory factors; as a result, it forms a vicious circle (129).

#### 3.1.2 Metabolic changes of T lymphocytes and their functional impact

T lymphocytes also undergo obvious metabolic reprogramming during activation and differentiation (130). Naive T cells have low metabolic activity and mainly rely on oxidative phosphorylation. After being activated by antigens, their metabolic activity increases sharply. They quickly switch to glycolysis to meet the energy and material needs of rapid proliferation (131).

Different T-cell subsets have different metabolic characteristics. Th1 and Th17 cells, which are pro-inflammatory, rely heavily on glycolysis and glutamine metabolism (132). Tregs, which are anti-inflammatory, mainly rely on oxidative phosphorylation and fatty acid oxidation (133). In the microenvironment of atherosclerotic plaques, the metabolic disorder of T cells is obvious. This disorder leads to the excessive activation of pro-inflammatory T cells and the dysfunction of Treg cells, which further promotes the progression of the disease (134).

If the metabolic reprogramming of immune cells can be reversed, it may become a new way to treat CHD. For example, using drugs to enhance oxidative phosphorylation of macrophages may promote their transformation to the M2 type, thereby stabilizing the plaque (135).

Metabolic reprogramming of immune cells is a core node in the inflammation-immune network of CHD. The dynamic balance between glycolysis and oxidative phosphorylation can link external stimuli, inflammatory responses, and plaque formation, laying the foundation for intervening and studying immune regulation through metabolic means. The key regulatory nodes of the MII network in CHD are shown in **Table 2**.

**Table 2:** Key regulatory nodes of the MII network in CHD.

Pathological state	Metabolic feature	Regulatory axis	Key cells	Biological function in plaque	References
Endothelial dysfunction	Enhanced glycolysis	mTOR–HIF-1α	Macrophages	Promote adhesion molecule expression and monocyte recruitment	Medina-Leyte DJ, 2021 (Int J Mol Sci) (136); Laaksonen R, 2016 (Eur Heart J) (137)
Foam cell formation	Hyper-glycolysis, suppressed OXPHOS	mTOR–HIF-1α	Macrophages	Boost lipid uptake and pro-inflammatory cytokine secretion	Laaksonen R, 2016 (Eur Heart J) (137); Moore KJ, 2013 (Nat Rev Immunol) (19)
Vulnerable plaque	Sustained high glycolysis	mTOR–HIF-1α	Macrophages	Increase matrix degradation and plaque instability	Moore KJ, 2013 (Nat Rev Immunol) (19); Hou P, 2023 (Cell Death Dis) (138)

ATVB, arteriosclerosis, thrombosis, and vascular biology; HIF-1α, hypoxia-inducible factor 1α; MII, metabolism-inflammation-immunity; mTOR, mechanistic target of rapamycin; OXPHOS, oxidative phosphorylation.

#### 3.1.3 Detection methods and clinical translation of immune cell metabolic reprogramming

The metabolic reprogramming of immune cells is a core regulatory node in the MII network of CHD, and its accurate detection and targeted intervention are critical for translational research. This section systematically elaborates on the multilevel detection methods of immune cell metabolic reprogramming and the therapeutic drugs/interventions that have entered clinical trials, providing a direct link between basic research and clinical application.

The detection of immune cell metabolic reprogramming is mainly carried out from three dimensions: cellular metabolic phenotype quantification, key metabolic molecule analysis, and *in vivo* non-invasive imaging, which complement each other to reflect the metabolic state of immune cells in atherosclerotic plaques and peripheral circulation. At the cellular level, the Seahorse XF Cell Energy Metabolism Analyzer is the gold standard for direct quantification of immune cell metabolic activity, which can detect the ECAR (extracellular acidification rate) and OCR (oxygen consumption rate) of macrophages and T lymphocytes in real time to reflect the levels of glycolysis and oxidative phosphorylation, respectively (139). Flow cytometry is widely used to detect the expression of key functional proteins in metabolic pathways [e.g., GLUT1 (glucose transporter 1), HK2 (hexokinase 2) for glycolysis; PPAR-γ (peroxisome proliferator-activated receptor γ), PGC-1α (peroxisome proliferator-activated receptor gamma coactivator 1α) for oxidative phosphorylation] on the surface or inside immune cells, so as to classify and quantify metabolically reprogrammed cell subsets. At the molecular level, qRT-PCR (quantitative real-time polymerase chain reaction) and Western blot are used to detect the mRNA and protein expression of core regulatory molecules in metabolic signaling pathways [e.g., mTOR-HIF-1α, STAT6 (signal transducer and activator of transcription 6)], and LC-MS (liquid chromatography-mass spectrometry) is applied to determine the content of metabolic intermediates (e.g., succinic acid, α-ketoglutarate) in immune cells, which can reflect the activation status of specific metabolic pathways and their regulatory effects on inflammatory phenotypes. At the *in vivo* level, PET (positron emission tomography) combined with ^18^F-FDG (^18^F-fluorodeoxyglucose) is a mature non-invasive imaging technology for *in vivo* detection of immune cell metabolic reprogramming. It can target and visualize the glycolysis activity of pro-inflammatory immune cells (M1 macrophages, Th1/Th17 cells) in atherosclerotic plaques by tracking glucose uptake and realize the quantitative evaluation of plaque metabolic activity and the dynamic monitoring of intervention efficacy.

With the deepening of research on the regulatory mechanism of immune cell metabolic reprogramming in CHD, a variety of targeted intervention strategies have entered phase I/II clinical trials, and the core targets focus on the key regulatory molecules and pathways of glycolysis and oxidative phosphorylation. These interventions have shown promising efficacy in reducing plaque inflammation and stabilizing vulnerable plaques. Metformin, a classic AMPK (AMP-activated protein kinase) activator, inhibits the mTOR-HIF-1α glycolysis pathway of macrophages, blocks the polarization of M1 pro-inflammatory phenotype, and promotes the transformation to M2 anti-inflammatory phenotype. A phase II clinical trial (NCT02965598) confirmed that metformin can significantly reduce the levels of systemic inflammatory markers [hs-CRP (high-sensitivity C-reactive protein) and IL-6 (interleukin-6)] in CHD patients and improve the stability of atherosclerotic plaques, with good safety and tolerability (140). Rapamycin, a specific mTOR inhibitor, can inhibit the excessive glycolysis of pro-inflammatory Th1/Th17 cells and enhance the oxidative phosphorylation of Treg, thereby restoring the immune balance of T-cell subsets in atherosclerotic plaques. A phase I clinical trial (NCT03382387) for ACS patients showed that low-dose rapamycin can reduce the recurrence rate of MACE (major adverse cardiovascular events) by regulating T-cell metabolism (141). Succinic acid receptor antagonists (e.g., GSK2578215A) block the succinic acid-mediated inflammatory activation of macrophages by inhibiting the SUCNR1 (succinate receptor 1) receptor and then reverse the metabolic reprogramming of macrophages and reduce plaque inflammation. This drug is currently in phase I clinical trial (NCT04251902) for CHD, and the preliminary results show that it can effectively reduce the glycolysis activity of peripheral blood monocytes in patients without obvious adverse reactions (142).

In summary, the multidimensional detection methods of immune cell metabolic reprogramming provide a reliable technical means for the early diagnosis and risk stratification of CHD, and the targeted interventions entering clinical trials open up a new direction for the precise treatment of CHD based on the MII network. However, most of the current interventions are still in the early clinical trial stage, and large-sample, multicenter phase III clinical trials are needed to further verify their long-term efficacy and optimal application population.

### 3.2 The central regulatory role of the inflammasome

The inflammasome is a multiprotein complex in the cytoplasm. It is an important part of the innate immune system and a key link connecting metabolic disorders and inflammatory responses (143). The activation of the inflammasome can trigger the maturation and release of pro-inflammatory cytokines such as IL-1β and IL-18 (interleukin-18), which play a key role in the occurrence and development of atherosclerosis (144).

#### 3.2.1 Activation mechanism of the NLRP3 inflammasome in coronary heart disease

Among all types of inflammasomes, the NLRP3 (NOD-like receptor family pyrin domain containing 3) inflammasome is the most studied and has the closest relationship with CHD (145). Its activation is a two-step process. The first step is the priming stage. Stimuli such as oxLDL and pro-inflammatory cytokines activate the NF-κB pathway, which increases the expression of NLRP3 and pro-IL-1β (146). The second step is the activation stage. Danger signals such as cholesterol crystals, ROS, and ATP activate the NLRP3 inflammasome, leading to the assembly of the complex (147).

In atherosclerotic plaques, cholesterol crystals are important activators of the NLRP3 inflammasome (148). These crystals are taken up by macrophages and cause lysosomal rupture. The released cathepsin B then activates the NLRP3 inflammasome (149). In addition, metabolic disorders such as high glucose and high fat can also induce the production of ROS, which further promotes the activation of the NLRP3 inflammasome (150).

#### 3.2.2 The inflammasome as a bridge connecting metabolism and inflammation

The NLRP3 inflammasome is like a sensor of metabolic disorders (151). It can recognize various metabolic stress signals in the body, such as excess cholesterol, high glucose, and free fatty acids. Once activated, it converts these metabolic signals into inflammatory responses, which is the core mechanism of the metabolism-inflammation-immunity network (152).

The activation of the inflammasome can not only directly promote the inflammatory response but also affect the metabolic reprogramming of immune cells (153). For example, IL-1β can inhibit the oxidative phosphorylation of macrophages and enhance glycolysis, which further stabilizes the pro-inflammatory phenotype of macrophages (154). This forms a positive feedback loop between inflammation and metabolism, making the pathological state of the plaque continue to deteriorate.

Clinical studies have shown that the level of IL-1β in the blood of patients with acute coronary syndrome is significantly increased (155). Blocking the IL-1β pathway can reduce the risk of cardiovascular events, which confirms the important role of the inflammasome in the clinical progression of CHD (156). However, several important controversies and limitations still exist for clinical translation. First, safety concerns remain prominent, including an increased risk of severe infection, neutropenia, and gastrointestinal adverse events associated with long-term IL-1β blockade, which restricts its broad clinical application (157). Second, standardized and evidence-based patient selection criteria are lacking; current trials include highly heterogeneous populations, making it difficult to identify subgroups that benefit most from anti-inflammatory therapy (158). Third, reliable and validated response biomarkers for monitoring therapeutic efficacy and predicting clinical outcomes are still insufficient, leading to inconsistent trial results and challenges in personalized treatment (159). These controversies and limitations highlight the need for further optimization of patient stratification, safer therapeutic strategies, and qualified biomarkers to improve the clinical utility of inflammasome-targeted interventions.

### 3.3 Interaction between metabolic hormones and immune inflammatory responses

Metabolic hormones not only are responsible for regulating energy metabolism but also play an important role in regulating immune and inflammatory responses (160). Abnormal secretion of these hormones is an important reason why metabolic diseases such as diabetes and obesity are closely related to CHD (161).

#### 3.3.1 Role of insulin and insulin resistance

Insulin is a key hormone regulating glucose metabolism. It also has anti-inflammatory effects under normal physiological conditions (162). It can inhibit the activation of macrophages and the secretion of pro-inflammatory cytokines and maintain the stability of the vascular endothelium (163).

However, in the state of insulin resistance, the body’s sensitivity to insulin decreases (164). To maintain normal blood glucose, the pancreas secretes more insulin, leading to hyperinsulinemia. At this time, the anti-inflammatory effect of insulin is weakened, and its pro-inflammatory effect is enhanced (165). It can promote the expression of adhesion molecules on the surface of endothelial cells and accelerate the adhesion and migration of immune cells (166).

Insulin resistance also leads to abnormal glucose metabolism of immune cells. Macrophages and T cells in a high-glucose environment are more likely to be activated and secrete pro-inflammatory factors, which further exacerbates atherosclerosis (167).

#### 3.3.2 Regulatory effect of adipokines on the network

Adipose tissue is not only a fat storage organ but also an important endocrine organ (168). It secretes a variety of adipokines, such as leptin, adiponectin, and resistin. These adipokines are important regulators of the metabolism-inflammation-immunity network (169).

Leptin is mainly secreted by white adipose tissue. It can regulate appetite and energy metabolism (170). At the same time, it can also activate immune cells, promote the secretion of pro-inflammatory factors, and accelerate the progression of atherosclerosis (171). In obese patients, the level of leptin in the blood is significantly increased, which is an important risk factor for CHD (172).

Adiponectin is a protective adipokine (173). It can improve insulin sensitivity, inhibit the activation of macrophages and the formation of foam cells, and reduce the inflammatory response (174). The level of adiponectin in patients with CHD is often decreased, and the lower the level, the more serious the disease (175).

The balance between pro-inflammatory adipokines and anti-inflammatory adipokines is crucial for maintaining vascular homeostasis. The imbalance of this balance is an important link connecting obesity, inflammation, and CHD (176).

### 3.4 Oxidative stress as a common pathway of the network

Oxidative stress refers to the imbalance between the production of ROS and the body’s antioxidant capacity (177). Reactive oxygen species are by-products of cellular metabolism. Under normal conditions, their level is low and they play a role in signal transduction. However, under pathological conditions, a large number of ROS are produced, which cause damage to cells and tissues (178).

Oxidative stress is a common pathway for the interaction of metabolism, inflammation, and immunity. It is both a product of network disorders and a driving force for network disorders, forming a complex regulatory network.

#### 3.4.1 Oxidative stress induced by metabolic disorders

Metabolic disorders are the main cause of oxidative stress. In the state of hyperlipidemia, oxLDL is produced in large quantities, which can directly induce the production of ROS in vascular cells (179). In diabetes, high glucose can activate the mitochondrial electron transport chain and the NADPH oxidase system, leading to a sharp increase in the level of ROS.

Free fatty acids can also induce oxidative stress. They promote the activation of NADPH oxidase in macrophages and vascular smooth muscle cells, resulting in the excessive production of ROS. These ROS further damage the structure and function of cells, exacerbating metabolic disorders (180).

#### 3.4.2 Oxidative stress regulates immune inflammatory responses

ROS are important signaling molecules in immune cells. They can activate the NLRP3 inflammasome and also promote the maturation and release of IL-1β (181). In addition, they can activate the NF-κB pathway, increase the expression of pro-inflammatory cytokines, and amplify the inflammatory response.

Oxidative stress can affect the metabolic reprogramming of immune cells (182). It inhibits the activity of mitochondrial enzymes and forces macrophages to switch to glycolysis. In turn, it promotes their polarization to the pro-inflammatory M1 type. At the same time, ROS cause DNA damage and immune cell apoptosis, and it leads to the accumulation of apoptotic cells in the plaque and the expansion of the necrotic core (183).

#### 3.4.3 Oxidative stress damages vascular homeostasis

ROS can directly damage the vascular endothelium. They oxidize and inactivate nitric oxide, and this reduces the vasodilation function of blood vessels (184); they also cause lipid peroxidation of the cell membrane, which leads to death of the endothelial cells and increased vascular permeability.

Oxidative stress can promote the proliferation and migration of vascular smooth muscle cells, and this leads to vascular wall thickening and plaque formation (185). It can also degrade the collagen fibers in the fibrous cap, which reduces plaque stability and increases the risk of rupture.

The MII network, in conclusion, is a complex and interconnected regulatory system. The four core links of this network are metabolic reprogramming of immune cells, activation of the inflammasome, regulation of metabolic hormones, and oxidative stress. They interact with each other, and together they promote the occurrence and development of CHD (186). Understanding the network is important for researchers to develop new treatment strategies for CHD.

### 3.5 Integrative crosstalk among epigenetic modifications

Although DNA methylation, histone modifications, and non-coding RNAs have been introduced separately in previous sections, these epigenetic regulatory mechanisms do not act in isolation. Instead, they form a highly coordinated and interdependent network that jointly determines chromatin structure and gene expression profiles during atherosclerosis progression (187).

DNA methylation often provides a foundational signal for the recruitment of histone-modifying enzymes, such as histone deacetylases and repressive histone methyltransferases, which further condense chromatin and reinforce transcriptional silencing. In contrast, permissive histone marks, including histone acetylation and active histone methylation, maintain an open chromatin conformation that inhibits *de novo* DNA methylation and enables the binding of transcription factors and non-coding RNAs (188).

Non-coding RNAs act as critical upstream and downstream regulators in this crosstalk. Many microRNAs and long non-coding RNAs directly target DNA methyltransferases, histone acetyltransferases, or histone deacetylases, thereby reshaping global DNA methylation and histone modification patterns. Reciprocally, DNA methylation and histone modifications control the transcription of non-coding RNAs, forming feedback loops that stabilize epigenetic states (189).

This cooperative interaction is particularly prominent in the regulation of key genes such as BDNF (brain-derived neurotrophic factor), whose transcriptional activity is fine-tuned by the combined effects of promoter DNA methylation, histone H3/H4 acetylation, and microRNA-mediated posttranscriptional silencing (190). In the setting of endothelial dysfunction, inflammation, and EndMT, such epigenetic crosstalk sustains pathological gene expression programs and accelerates atherosclerotic development.

Therefore, an integrative understanding of epigenetic cooperation is essential for elucidating the molecular mechanisms of atherosclerosis and designing more effective epigenetic-targeted interventions.

## 4 Risk factors and susceptibility mechanisms of coronary heart disease

The occurrence and progression of CHD are not just simple local vascular pathological changes; they are also affected by various risk factors. For example, genetic susceptibility, metabolic abnormalities, lifestyle, and environmental factors all play a role in the occurrence and progression of CHD. Understanding the molecular mechanisms behind these pathogenic factors can help us find key methods of intervention, and this provides a scientific basis for personalized treatment.

### 4.1 Common pathogenic factors and their molecular basis

There are many traditional pathogenic factors for CHD, especially hyperlipidemia, hypertension, diabetes, smoking, drinking, and lack of exercise. These factors affect vascular endothelial function, activate inflammatory responses, interfere with lipid metabolism and immune regulation through various molecular mechanisms, and ultimately lead to atherosclerosis and increase the risk of CHD.

#### 4.1.1 Mechanisms of action of pathogenic factors such as hyperlipidemia, hypertension, and diabetes

Hyperlipidemia, hypertension, and diabetes are traditional pathogenic factors of CHD. They all accelerate the process of atherosclerosis by inducing endothelial damage, activating inflammatory responses, and regulating cell functions and other pathways; there is also a mutual positive feedback effect between the three, which jointly promotes the occurrence and development of CHD.

Hyperlipidemia is the main factor leading to coronary atherosclerosis, among which the increase of LDL has the most critical impact. Excessive LDL will deposit in areas with vascular endothelial damage and then be oxidatively modified into oxLDL and then recognized and phagocytized by scavenger receptors CD36 and SR-A on the surface of macrophages. This will transform macrophages into foam cells and at the same time promote macrophages to release pro-inflammatory factors IL-1β and TNF-α, amplifying the local inflammatory response. In addition, high levels of LDL can also promote endothelial cells to produce ROS, inhibit the synthesis of nitric oxide, damage the normal diastolic function of blood vessels, and further aggravate vascular endothelial damage (191).

Hypertension increases the shear stress and mechanical load of the vascular wall, leading to endothelial dysfunction and promoting the development of CHD. After the vascular wall is stimulated by mechanical stretching, it will induce the response of endothelial cells, express adhesion molecules VCAM-1 (vascular cell adhesion molecule-1) and ICAM-1 (intercellular adhesion molecule-1), and activate oxidative stress response, making it easier for monocytes to adhere to endothelial cells and migrate to the vascular intima (192). In addition, hypertension can directly activate vascular smooth muscle cells, promote the proliferation and migration of smooth muscle cells, accelerate the formation of fibrous caps, promote vascular sclerosis, and ultimately promote the expansion of atherosclerotic plaques.

Diabetes accelerates atherosclerosis through multiple synergistic pathways, which is also an important risk factor for CHD. Under the state of long-term hyperglycemia, a large number of AGEs (advanced glycation end products) will be formed in the human body. AGEs bind to RAGE (receptor for advanced glycation end products) receptors on the cell surface, activate the NF-κB signaling pathway, promote the continuous secretion of inflammatory factors, and amplify the inflammatory response; at the same time, the high-glucose environment in the body will induce macrophages to polarize to the pro-inflammatory phenotype (M1 type), enhance the glycolytic activity in cells, and further strengthen the pro-inflammatory response; high blood sugar will also aggravate oxidative stress, inhibit the activity of eNOS, damage the integrity of vascular endothelium, and accelerate the formation of foam cells. Therefore, diabetes promotes atherosclerosis from multiple dimensions (193).

Hyperlipidemia, hypertension, and diabetes are often accompanied by systemic metabolic abnormalities, and they also jointly activate the inflammatory regulatory network, with complex interactions between the three (194). The three synergistically promote oxidative stress response, activate immune cells, and aggravate vascular endothelial damage, thereby jointly accelerating the occurrence and development of CHD and making the condition more serious.

#### 4.1.2 Effects of smoking, drinking, and lack of exercise on coronary heart disease

Smoking, drinking, and lack of exercise are key factors leading to CHD in daily life. They affect vascular function by regulating blood lipid metabolism, oxidative stress, inflammatory response, and other pathways, thereby inducing CHD. Moreover, such factors are usually controllable. Therefore, maintaining a healthy lifestyle is an important way to prevent and control CHD (195).

Smoking is harmful to CHD in many ways. Tobacco contains many harmful components such as nicotine and free radicals, which directly attack the vascular endothelium, damage its integrity, and at the same time promote the massive production of ROS, inducing the increased secretion of pro-inflammatory factors and thereby amplifying the local inflammatory response; in addition, smoking activates platelet function and significantly increases the degree of platelet activation, making thrombosis more likely to form, and smoking reduces the level of HDL, interfering with the process of reverse cholesterol transport. Under such a dual effect, the formation of atherosclerotic plaques is accelerated, further increasing the incidence of acute CHD (196).

The impact of drinking on the cardiovascular system is closely related to the amount of drinking, and drinking is not absolutely harmful or beneficial. Moderate drinking, especially moderate red wine drinking, can have a certain protective effect on the cardiovascular system. This protective effect is mainly achieved by increasing HDL levels, inhibiting abnormal platelet aggregation, and exerting a mild anti-inflammatory effect (197). However, long-term and heavy drinking will break this protective effect but lead to increased blood pressure, exacerbate the pro-inflammatory response and oxidative stress state in the body, damage vascular endothelial function, and at the same time interfere with lipid metabolism, ultimately accelerating atherosclerosis and increasing the risk of CHD.

Lack of exercise promotes the development of CHD by directly damaging vascular function and indirectly inducing metabolic disorders; insufficient exercise is likely to cause obesity, metabolic syndrome, insulin resistance, and other problems, leading to abnormal lipid profile, increased levels of LDL and triglycerides, and decreased HDL levels. Moreover, lack of exercise will weaken the diastolic capacity of blood vessels, increase the level of inflammatory factors in the body, and reduce the activity of antioxidant enzymes, reducing the body’s ability to clear oxidative products. Therefore, long-term lack of exercise will continuously aggravate vascular damage and accelerate the occurrence and progression of atherosclerosis (198).

Unhealthy lifestyles such as smoking, drinking, and lack of exercise have become important factors in the occurrence and progression of CHD by affecting three key pathways: blood lipid metabolism, oxidative stress, and inflammatory response. For such lifestyles, in the treatment process, targeted intervention plans can be formulated in combination with individual genetic material and metabolic status. This therapy can provide a solid scientific basis for the individualized prevention and treatment of CHD.

### 4.2 New pathogenic factors and systemic inflammatory states

In addition to traditional pathogenic factors such as smoking, drinking, and lack of exercise, some new factors such as environmental pollution, psychological stress, sleep disorders, and intestinal flora imbalance have also been confirmed to play an important role in the induction of CHD. These factors usually activate systemic inflammatory responses, induce metabolic disorders and immune dysfunction, and then accelerate atherosclerosis. Such new factors also provide new explanations for the complex etiology of CHD.

#### 4.2.1 Effects of environmental pollution, psychological stress, sleep disorders, and other factors on CHD

Airborne fine PM2.5 (particulate matter 2.5), heavy metals such as lead and mercury, and other pollutants can enter the whole body through pulmonary circulation and blood circulation, directly inducing vascular endothelial damage and triggering oxidative stress; among them, PM2.5 can also activate the NLRP3 inflammasome of macrophages, increase the secretion of inflammatory factors IL-1β and TNF-α, and form a low-grade chronic inflammatory environment in the body; at the same time, PM2.5 can also lead to increased blood pressure and decreased heart rate variability, which will increase the risk of acute myocardial infarction and have an important impact on the induction of CHD (199).

Long-term psychological stress and chronic anxiety will activate the HPA (hypothalamic–pituitary–adrenal) axis and sympathetic nervous system, which will increase the levels of stress hormones such as catecholamines and glucocorticoids in the body. High levels of stress hormones will induce immune cells to polarize to the pro-inflammatory phenotype, secrete IL-6 and TNF-α, and at the same time promote the proliferation of vascular smooth muscle cells, accelerate the deposition of oxLDL, and ultimately accelerate the process of atherosclerosis. Therefore, this is also an important inducement of CHD (200).

Chronic sleep deprivation or sleep disorders caused by OSA (obstructive sleep apnea) can lead to increased blood pressure, metabolic abnormalities, and systemic inflammation. Such sleep disorders will increase sympathetic nerve activity, promote ROS production, activate NF-κB, enhance the inflammatory response of vascular endothelium, and form a multidimensional network associated with insulin resistance, obesity, and lipid metabolism, which jointly promote the development of CHD (201).

Environmental, psychological, and sleep factors interact through systemic inflammation, oxidative stress, and metabolic imbalance to jointly induce CHD. This also reasonably explains why some patients develop early or severe CHD even if their lifestyle is relatively healthy.

#### 4.2.2 Association between intestinal flora and coronary heart disease

Intestinal flora imbalance has been confirmed to be a key factor inducing CHD. It mainly plays a role in the body by regulating metabolites and activating inflammatory responses and immune regulation mechanisms. Intestinal flora can decompose food to produce a variety of metabolites, among which TMA (trimethylamine), TMAO (trimethylamine oxide), and short-chain fatty acids (SCFAs) have obvious effects on the process of CHD. The increase of the TMAO level is closely related to atherosclerosis, platelet activation, and thrombosis, whereas SCFAs such as acetic acid and butyric acid have anti-inflammatory effects, which can improve immune homeostasis and endothelial function through G protein-coupled receptors and HDAC (histone deacetylase) inhibition, thus forming a two-way regulation at the metabolic level (202).

When the intestinal barrier function is damaged or the intestinal flora is imbalanced, LPS (endotoxin) will enter the blood circulation, activate the Toll-like receptor 4 (TLR4) signaling pathway, and then induce the activation of NF-κB and NLRP3 inflammasome, triggering systemic low-grade inflammation. This long-term chronic inflammatory state will not only accelerate the formation of foam cells but also affect the ratio of Treg cells/Teff cells, leading to the destruction of MII balance and further promoting the development of CHD (203).

In recent years, a number of clinical studies have confirmed that the level of TMAO is positively correlated with the severity of CHD, the instability of plaques, and the risk of recurrent myocardial infarction, which highlights the close association between intestinal flora and CHD (204). Regulating intestinal flora through probiotic intervention, dietary adjustment, or targeted metabolite intervention can effectively improve the body’s inflammatory state and regulate blood lipid profile, which has potential application value for the prevention and auxiliary treatment of CHD.

As a new type of risk factor, intestinal flora interacts with traditional pathogenic factors such as smoking, drinking, and lack of exercise through systemic inflammation and metabolic-immune regulatory networks, affecting the occurrence of CHD, plaque formation, and the incidence of acute CHD, providing an important theoretical basis for the treatment of CHD (205).

### 4.3 Genetic factors and the occurrence of coronary heart disease

Not only is the occurrence of CHD affected by environmental and lifestyle factors, but also genetics is an important determinant. In recent years, genomic studies have found a large number of genetic variants related to CHD, which provides a new perspective for studying individual susceptibility to CHD and formulating early intervention therapies for CHD. PRS (polygenic risk score) can effectively assess an individual’s risk of CHD by combining multiple genetic variants, but it still faces many challenges in practical clinical applications.

#### 4.3.1 Relationship between genetic susceptibility to coronary heart disease and family history

A large number of epidemiological studies have shown that if there are patients with early-onset CHD in a family, the individual’s risk of developing the disease will increase significantly. Among them, having a first-degree relative with CHD will increase the individual’s risk of developing CHD by two to three times. This conclusion fully indicates that genetic factors play an important role in the pathogenesis of CHD; in addition, family history not only is affected by genes but also may be affected by similar lifestyles among family members. These factors are intertwined and jointly affect the risk of CHD.

In GWAS (genome-wide association studies), a variety of SNPs (single-nucleotide polymorphisms) have been found to be closely related to CHD, among which the most common are variations in the 9p21 region and variations in genes such as LDLR (low-density lipoprotein receptor), APOB (apolipoprotein B), PCSK9 (proprotein convertase subtilisin/kexin type 9), and SORT1 (sortilin 1). These genes are widely involved in lipid metabolism, maintaining vascular endothelial function and regulating inflammatory responses and thrombosis, which are closely related to the pathogenesis of CHD. Taking the variation at the 9p21 locus as an example, the variation at 9p21 can regulate the proliferation process of smooth muscle cells by affecting the expression of CDKN2A/2B (cyclin-dependent kinase inhibitor 2A/2B) genes, thereby accelerating the progression of atherosclerosis (206).

The genetic susceptibility to CHD is not a single mode but presents the characteristics of frequency-effect polymorphism; that is, genetic variants with different frequencies regulate disease risk through synergistic effects, forming a complex genetic architecture. Common variants (allele frequency >5%) such as CDKN2A/CDKN2B gene variants in the 9p21 region, although the effect of a single variant on the risk of CHD is weak (ratio approximately 1.29), have a high carrying rate in the population, making them important genetic drivers of CHD at the population level. Their mechanism may accelerate atherosclerosis by inhibiting the interferon-γ signaling pathway and promoting the proliferation of vascular smooth muscle cells; low-frequency variants (1:1,000~1:20) and rare variants (<1:1,000) such as loss-of-function mutations in genes such as PCSK9, LDLR, and APOB, although the carrying rate is low, can have a strong protective or pathogenic effect by significantly affecting lipid metabolism [such as PCSK9 mutations reducing LDL-C (low-density lipoprotein cholesterol) levels]. Among them, carriers of rare PCSK9 mutations have a 40% lower risk of CHD than the general population (207). Mendelian randomization studies have further confirmed that LDL-C and lipoprotein a-related genetic variants are causally associated with CHD, whereas CRP (C-reactive protein) gene variants have no direct causal relationship with CHD, clarifying the core role of lipid metabolism pathways in the disease. In addition, the phenotypic complexity of genetic variants is significant. For example, different mutations in the SCN5A (sodium voltage-gated channel alpha subunit 5) gene can lead to long QT syndrome, Brugada syndrome, and dilated cardiomyopathy, reflecting the pleiotropy of genes; the clinical phenotypic heterogeneity of Marfan syndrome caused by FBN1 (fibrillin-1) gene mutations is related to the penetrance and expressivity jointly affected by modifier genes and environmental factors (208).

Genetic factors of CHD do not play a role in isolation but are closely related to environmental factors. Unhealthy lifestyles such as hyperlipidemia, hypertension, and smoking often amplify the genetic risk of the disease. In recent years, some clinical studies have found that individuals carrying high-risk genotypes for CHD have a significantly higher probability of early-onset CHD when they have bad living habits such as smoking and high-fat diet. This also confirms that the prevention of CHD should start from both genetic and environmental factors.

#### 4.3.2 Application and challenges of polygenic risk score

Polygenic risk score can integrate the risk effects of dozens to millions of SNPs to generate a single continuous risk score, so as to assess each individual’s genetic tendency to CHD (209). Therefore, this scoring system has obvious stratification. Among them, individuals with high PRS may have a higher risk of CHD even if their lifestyle is relatively healthy and traditional pathogenic factors are not obvious; on the contrary, some individuals with low PRS may still have a relatively lower risk of CHD when facing certain environmental pressures. Thus, PRS provides a more accurate quantitative tool for assessing the genetic risk of CHD.

PRS has multiple application values in the clinical field. PRS can be used for early risk identification, which can accurately lock in high genetic risk groups in adolescence or early adulthood and provide targeted early prevention for this group, scientifically and reasonably adjust their lifestyle, and reduce the incidence rate from the source. At the same time, PRS can provide a basis for personalized treatment. In recent years, clinical studies have shown that patients with high PRS often have more obvious curative effects when using statins or PCSK9 inhibitors. In addition, combining PRS with traditional pathogenic factors for analysis can optimize the method of screening CHD populations, further reduce the burden of CHD patients when they develop the disease, and improve public health level to a certain extent.

Although the application prospect of PRS is very broad, there are still many challenges and limitations in the practical application of PRS. Differences between races and populations are one of the core issues. At present, most PRSs are constructed based on GWAS of European populations. When applied to other populations such as Asians and Africans, the accuracy of predicting the incidence of CHD will be significantly reduced, making it difficult to meet the risk assessment needs of diverse populations. In terms of risk, PRS can only reflect the genetic risk. It does not consider key factors such as the lifestyle, metabolic status, and environment of different populations, and it still needs to be further combined with multidimensional clinical information to improve the scientificity of the assessment (210). At the same time, ethical and privacy issues cannot be ignored. The results of genetic risk assessment may bring psychological pressure to the assessed person and may also trigger a series of problems such as leakage of genetic data privacy. Therefore, we should establish a sound application specification and management system and cautiously promote the clinical application of PRS.

**Figure 5:**
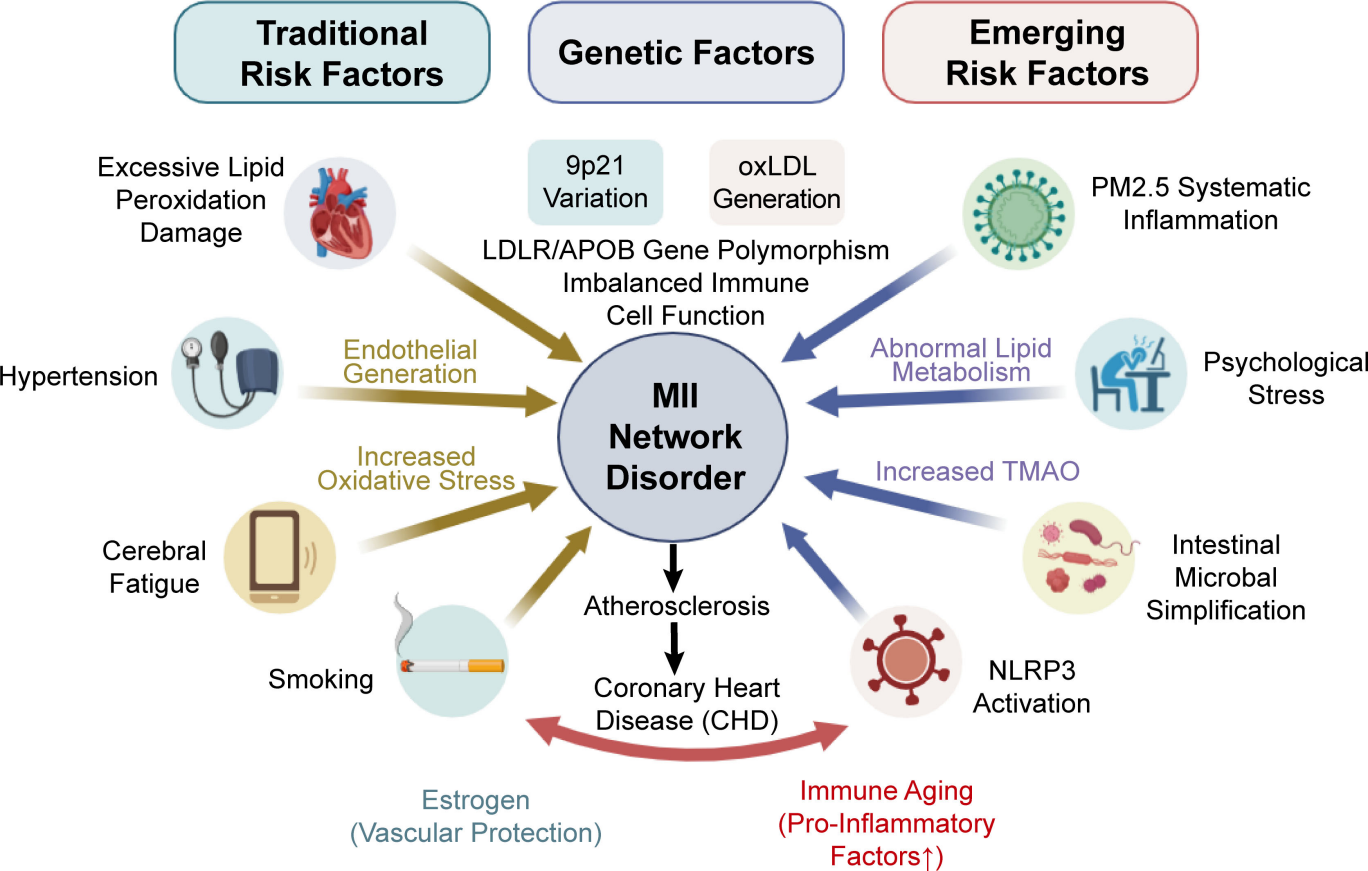
Integrated mechanism diagram of risk factors and susceptibility regulation in CHD.

In general, both genetic factors and PRS provide important ways for predicting the risk of CHD, but in clinical practice, genetic factors and PRS still need to be further combined with traditional and new pathogenic factors, as well as metabolic-inflammatory networks, so as to build a comprehensive risk assessment system. Only in this way can we more accurately guide the prevention, diagnosis, and treatment of CHD in clinical work. The relationship between the pathogenic factors of CHD and the susceptibility regulation mechanism is illustrated in **Figure 5**.

### 4.4 Gender differences and age-related mechanisms

The occurrence and progression of CHD are not only affected by traditional and new pathogenic factors but also show significant gender and age differences. These differences involve four aspects: hormone regulation, vascular function, immune response, and metabolic status, explaining the differences in onset age, clinical manifestations, and disease progression characteristics between men and women.

#### 4.4.1 Gender differences in the pathogenesis of coronary heart disease

There are significant differences in the regulation of CHD risk between men and women by sex hormones. Among them, estrogen plays an important vascular protective role before menopause in women, promoting the production of nitric oxide, enhancing antioxidant capacity, and thereby inhibiting inflammatory responses. In addition, estrogen can also regulate blood lipid profile and increase HDL and decrease LDL levels, effectively reducing the incidence of CHD (211). Men have relatively high testosterone levels. Although testosterone helps blood vessel relaxation at an appropriate concentration, excessive testosterone or abnormal testosterone metabolism can promote lipid deposition and induce inflammatory responses and platelet activation, thereby increasing the risk of early-onset CHD.

There are inherent differences in vascular structure and function between men and women, thus affecting the onset characteristics and progression mode of CHD. For men, the coronary arteries are more prone to extensive plaque accumulation, and the formed plaques are mostly lipid-rich, with relatively higher instability, whereas CHD in women is more manifested as microvascular dysfunction and impaired endothelium-dependent diastolic function. Although the plaque stability of women is higher than that of men, the microvascular perfusion is more limited, which has also become an important pathological feature of CHD in women (212).

There are differences in immune and inflammatory responses between men and women, which further promote the differentiation of CHD between the two. The overall immune system activity of women is relatively higher, and the balance mechanism between pro-inflammatory and anti-inflammatory of macrophages and T cells is more complex. Estrogen can induce the polarization of M2 macrophages and inhibit the activation of NLRP3 inflammasome, thereby effectively reducing the inflammatory response in blood vessels, whereas men are more likely to have M1 macrophages dominate under stress or metabolic disorders, which will exacerbate the inflammatory response, increase the instability of plaques, and thus increase the incidence of acute CHD.

The clinical manifestations of CHD also show obvious differences between men and women, which also has an important impact on clinical diagnosis. Men are more likely to have typical angina pectoris and acute myocardial infarction symptoms, which also makes the identification and intervention of early CHD more convenient, whereas women often show atypical symptoms such as chest tightness, fatigue, and dyspnea, which are more likely to be ignored or misdiagnosed, leading to delayed diagnosis. These gender differences indicate that in the prevention and treatment of CHD, the differences in sex hormones, vascular structure characteristics, and immune responses should be fully considered, so as to formulate personalized prevention and treatment methods for CHD.

#### 4.4.2 Effects of age on the progression of coronary heart disease

With the growth of age, the structure and function of human blood vessels will undergo natural degenerative changes. The elasticity of the arterial wall gradually decreases, and the vascular stiffness increases continuously, which will lead to increased blood pressure. At the same time, the function of vascular endothelium will also be gradually damaged. This age-related vascular sclerosis and fibrosis change will make atherosclerotic plaques more likely to form, and the stability of plaques will be significantly reduced, ultimately increasing the risk of acute CHD (213). This is also an important physiological basis for the development of CHD.

Immune system aging, also known as immunosenescence, is also a key factor leading to CHD. With the growth of age, the function of immune cells will gradually decline, which is specifically manifested as the decrease of T-cell diversity, the decrease of macrophage clearance capacity, and the continuous increase of the levels of pro-inflammatory factors such as IL-6, TNF-α, and CRP, thus forming a state of chronic low-grade inflammation, that is, inflammaging. This long-term inflammatory microenvironment will continuously promote atherosclerosis, thereby accelerating the course of CHD (214).

The growth of age will also lead to the accumulation of metabolic disorders. Insulin resistance, abnormal lipid metabolism, and energy metabolism imbalance are more common in the elderly. At the same time, the mitochondrial function of the elderly will continue to decline, and the oxidative stress response will be enhanced. These factors are superimposed with chronic inflammation, which will further accelerate the formation of foam cells, make the fibrous cap on the plaque degenerate continuously, make the plaque more vulnerable to damage, and thus aggravate the progression of CHD from the metabolic level.

As one of the most influential independent pathogenic factors for CHD, age is particularly closely related to clinical practice. Elderly patients with CHD are often accompanied by a variety of coexisting risk factors, the characteristics of plaques are more complex, vascular dysfunction is more obvious, and their tolerance to surgery and drugs is relatively poor, leading to generally worse treatment effects than young patients. Overall, gender and age significantly change the clinical manifestations and disease progression of CHD by affecting vascular structure, endothelial function, immune response, and metabolic status. Therefore, in clinical management, these two factors should be incorporated into the risk assessment model, which can effectively provide a basis for the precise prevention and treatment of CHD.

## 5 New progress in diagnostic technology and risk assessment

The diagnosis of CHD shifts from traditional anatomical stenosis detection, to functional and pathological assessment. New technologies can now identify early vascular damage, unstable plaques, and systemic metabolic-inflammatory disorders. These advances can help doctors find high-risk patients earlier and make more accurate treatment plans. In addition, risk assessment is also improving (215), where it now combines traditional risk factors, genetic information, and molecular markers. The comprehensive approach makes risk prediction more personalized and reliable.

### 5.1 Progress in imaging diagnosis technology

Imaging is key to CHD diagnosis. New imaging methods not only show the location and degree of coronary stenosis but also evaluate plaque stability and myocardial blood supply; this helps doctors distinguish between stable and high-risk patients.

#### 5.1.1 Coronary computed tomography angiography

CCTA (coronary computed tomography angiography) is a non-invasive imaging method; it uses X-rays and computer processing to create clear 3D images of coronary arteries, and this can directly show whether there is stenosis and its severity. Recent improvements in technology have made images clearer and reduced radiation exposure (216). Thus, CCTA also helps identify plaque types; it can tell the difference between calcified plaques and non-calcified plaques. Non-calcified plaques are often more unstable and have a higher risk of rupture. In recent studies, researchers discover that CCTA is accurate in ruling out CHD; for example, negative CCTA result means a very low risk of a heart attack in the short term (217). However, CCTA has its limits; it cannot clearly show the function of blood vessels or the actual blood flow to the heart muscle, and it also has trouble evaluating severely calcified arteries.

#### 5.1.2 Intravascular imaging technology

Intravascular imaging is used in coronary angiography. It provides real-time, high-resolution images from inside the blood vessels, and this helps doctors see plaque details and make better decisions during surgery. IVUS (intravascular ultrasound) uses sound waves to create images of the vessel wall and plaque (218). It can measure the size of the plaque and the thickness of the vessel wall, and this is useful for guiding stent placement. OCT (optical coherence tomography) uses light waves, and it has even higher resolution than IVUS. OCT can clearly show the thin fibrous cap of vulnerable plaques, cholesterol crystals, and thrombus; currently, it is the best tool available to identify plaque rupture and erosion (219). The main limit of intravascular imaging is that it only focuses on small sections of the arteries, and it also requires inserting a catheter into the blood vessel, which adds up risk potential.

#### 5.1.3 Myocardial perfusion imaging and functional assessment

Myocardial perfusion imaging (MPI) is able to check whether the heart muscle is getting enough blood, and it can find functional problems even when coronary arteries are not showing obvious stenosis. Stress perfusion cardiac magnetic resonance (CMR) uses a magnetic field and radio waves (220). It can show blood flow in the heart muscle at rest and under stress and accurately identify areas of myocardial ischemia. Also, it can tell the differences between reversible ischemia and irreversible myocardial damage. PET uses small amounts of radioactive tracers (221). It can assess both blood flow and myocardial metabolism, and it is very helpful to discern if a portion of the heart muscle is still alive and could benefit from revascularization surgery.

These functional imaging methods are especially important for patients with non-obstructive coronary artery disease, and they can explain why patients have symptoms even with normal-looking arteries.

### 5.2 Biomarkers and molecular diagnosis

Biomarkers are substances in the blood that can indicate specific diseases (222). Some new biomarkers currently can reflect endothelial damage, inflammation, and plaque instability, and they provide important information for early diagnosis and risk stratification.

#### 5.2.1 Traditional cardiac biomarkers

CTn (cardiac troponins) are the best standard for diagnosing acute myocardial infarction. Hs-cTn (high-sensitivity cardiac troponin) tests, for example, can detect very low levels of troponin in the blood, and this allows for earlier and more accurate diagnosis of heart damage. CK-MB (creatine kinase MB) and myoglobin are also used in such diagnoses, but compared with troponins, they are less specific and are sometimes used together with troponins to confirm a diagnosis (223). These traditional biomarkers mainly reflect myocardial necrosis, and they cannot predict or detect early-stage CHD before a heart attack occurs.

Traditional cardiac biomarkers are mainly used for the diagnosis of acute myocardial necrosis, whereas with the in-depth research on the pathogenesis of CHD, a variety of biomarkers related to inflammation, plaque instability, and metabolic disorder have been discovered and applied in clinical practice. According to the pathological mechanism and clinical application value, CHD-related biomarkers can be divided into six categories, and each type of biomarker has unique characteristics in the diagnosis and prognosis of CHD. Classification of biomarkers for CHD diagnosis and prognosis is shown in **Table 3**.

**Table 3:** Classification of biomarkers for CHD diagnosis and prognosis.

Biomarker category	Core subtypes	Representative markers	Clinical application	Characteristic advantages	Limitations	References
Cardiac necrosis Biomarkers	Myocardial injury-specific markers	Cardiac troponin (cTnI/cTnT), hs-cTn, CK-MB, myoglobin	Diagnosis of AMI, risk stratification of ACS	High specificity/sensitivity for myocardial necrosis; hs-cTn enables early AMI diagnosis	Cannot detect early CHD before myocardial injury; CK-MB/Myoglobin with poor tissue specificity	Mach F, 2023 (Rev Med Suisse) (224); Libby P, 2021 (J Am Coll Cardiol) (225)
Inflammatory biomarkers	Systemic/plaques local inflammation markers	Hs-CRP, IL-6, TNF-α, MPO	Predict long-term cardiovascular event risk; evaluate plaque inflammatory activity; monitor anti-inflammatory therapy efficacy	Reflect systemic low-grade inflammation; correlate with plaque instability	Non-specific (elevated in other inflammatory diseases); cannot indicate lesion location	Ridker PM, 2017 (N Engl J Med) (152); Abbate A, 2020 (Circ Res) (158)
Plaque instability biomarkers	Fibrous cap degradation/plaque vulnerability markers	Matrix metalloproteinases (MMPs-1/9), PAPP-A, oxidized low-density lipoprotein (oxLDL)	Identify vulnerable plaques; predict plaque rupture risk; early warning of ACS	Directly reflect pathological changes of atherosclerotic plaques; specific for plaque instability	Low detection sensitivity in early stage; lack of unified clinical reference range	Naghavi M, 2003 (Circulation) (55); Hou P, 2023 (Cell Death Dis) (138)
Metabolic biomarkers	Metabolic disorder/gut flora-related markers	TMAO, adiponectin, leptin, Lp(a), TG	Evaluate systemic metabolic state; predict CHD risk in metabolic disease patients; reflect gut flora imbalance	Correlate with core MII network disorder; predict long-term CHD risk	Affected by diet/medication; individual variability is large	Laaksonen R, 2016 (Eur Heart J) (137); Moore KJ, 2013 (Nat Rev Immunol) (19)
Endothelial dysfunction biomarkers	Vascular endothelial injury/dysfunction markers	ET-1 (endothelin-1), vascular cell adhesion molecule-1 (VCAM-1), ICAM-1, nitric oxide (NO)	Detect early vascular endothelial injury; evaluate vascular homeostasis; predict atherosclerosis initiation	Reflect pre-plaque pathological changes; early CHD screening	Low stability in peripheral blood; detection methods are not unified	Medina-Leyte DJ, 2021 (Int J Mol Sci) (136); Katsiki N, 2014 (Curr Med Res Opin) (226)
Genetic biomarkers	Gene variants/polygenic markers	SNPs in 9p21 region, LDLR/APOB/PCSK9 gene variants, polygenic risk score (PRS)	Identify genetic susceptibility to CHD; personalized risk assessment; guide targeted lipid-lowering therapy	Reflect innate CHD risk; provide long-term risk prediction	Cannot reflect acquired risk factors; limited applicability in different races	Libby P, 2019 (Nat Rev Dis Primers) (211); Hsu PY, 2022 (Am J Cancer Res) (227)

ACS, acute coronary syndrome; AMI, acute myocardial infarction; CHD, coronary heart disease; CK-MB, creatine kinase MB; cTnI, cardiac troponin I; cTnT, cardiac troponin T; ET-1, endothelin-1; hs-CRP, high-sensitivity C-reactive protein; hs-cTn, high-sensitivity cardiac troponin; LDLR, low-density lipoprotein receptor; Lp(a), lipoprotein(a); MII, metabolism-inflammation-immunity; MPO, myeloperoxidase; PAPP-A, pregnancy-associated plasma protein A; PCSK9, proprotein convertase subtilisin/kexin type 9; PRS, polygenic risk score; SNPs, single-nucleotide polymorphisms; TG, triglyceride.

#### 5.2.2 New biomarkers for inflammation and plaque instability

Inflammatory biomarkers are becoming more and more important these days, and they can show the activity of the disease process. For example, high-sensitivity C-reactive protein (hs-CRP) is a widely used inflammatory marker. A high hs-CRP level means a higher long-term risk of cardiovascular events, even in people without obvious symptoms. Other kinds of new biomarkers include substances like IL-6, TNF-α, and MPO (myeloperoxidase), and these markers are closely related to inflammatory responses in atherosclerotic plaques (228).

Plaque instability biomarkers include MMPs and PAPP-A (pregnancy-associated plasma protein A), and these substances are released when the plaque’s fibrous cap is being damaged, indicating a high risk of rupture.

#### 5.2.3 Metabolic and genetic biomarkers

Metabolic biomarkers reflect the systemic metabolic disorders in CHD. For example, TMAO is a metabolite produced by intestinal bacteria, and high TMAO levels are linked to an increased risk of thrombosis and heart attacks. Adipokines such as adiponectin and leptin are also used as metabolic biomarkers, and low adiponectin levels are associated with more severe atherosclerosis. Genetic biomarkers, such as specific gene variants, can identify people with a high genetic risk of CHD (229). Combining genetic biomarkers with other risk factors can significantly improve the accuracy of risk prediction.

### 5.3 New progress in risk assessment models

Risk assessment models can combine multiple factors to estimate a person’s chance of developing CHD, or having a cardiovascular event (230). New models are now more comprehensive and accurate than the older ones.

#### 5.3.1 Improvement of traditional risk assessment models

Traditional models, such as the Framingham Risk Score and the SCORE (Systematic Coronary Risk Evaluation) system, are based on age, gender, blood pressure, cholesterol levels, smoking status, and diabetes (156). They are useful for assessing population risk, but lack precision for individual patients.

New versions of these models have been updated these years. They include more detailed factors, such as family history and kidney function, and they also use data from more diverse people to improve their range of adaptation. However, these models still have their defects, for they cannot fully capture the complex interactions between genes, metabolism, and inflammation (231).

#### 5.3.2 Introduction of polygenic risk score and integrated assessment

The Polygenic Risk Score (PRS) is a new tool, and it can calculate the cumulative genetic risk from thousands of gene variants (232). As is mentioned earlier, a high PRS means a significantly higher risk of early-onset CHD.

The most promising approach is integrated risk assessment, and it combines PRS, traditional risk factors, and molecular biomarkers (233). For example, a person with a high PRS, high hs-CRP, and abnormal lipid levels would be identified as a very high-risk patient, and the patient would need intensive preventive treatment.

Integrated assessment can also be used after treatment. After the treatment, it can monitor whether the treatment is effectively reducing the patient’s overall risk.

#### 5.3.3 Clinical application of risk stratification

Risk stratification refers to the process of dividing patients into different risk groups (234). In the process, it guides treatment decisions.

As for low-risk patients, they may only need lifestyle changes, and moderate-risk patients may need medication such as statins; for high-risk patients, they indeed need more aggressive treatment, including close monitoring, and possibly invasive procedures (235).

In acute coronary syndrome, rapid risk stratification is critical, and it helps doctors decide whether a patient needs urgent angiography and revascularization (123). Tools like the GRACE (Global Registry of Acute Coronary Events) score are usually used to assess in-hospital and long-term mortality risks in these patients.

The progress in diagnosis and risk assessment, in summary, allows for a more precise and personalized approach to CHD. By combining advanced imaging, multiple biomarkers, and genetic information, doctors can now detect the disease earlier, predict risks more accurately, and choose the most effective method of treatment for each patient.

#### 5.3.4 Integrated imaging and biomarker strategies for atherosclerosis diagnosis and prognosis

The combination of non-invasive imaging and circulating biomarkers enables precise risk stratification, early diagnosis, and prognostic evaluation of atherosclerotic cardiovascular disease, which has become a core development direction of modern CHD (CHD) diagnostic technology. Imaging modalities such as CCTA, ^18^F-fluorodeoxyglucose positron emission tomography (^18^F-FDG PET), and CMR provide direct anatomical morphology, metabolic activity, and functional perfusion information of atherosclerotic plaques, whereas circulating biomarkers including inflammatory factors, metabolic indicators, and plaque instability-related molecules reflect the systemic biological activity of CHD and the pathological state of local lesions (236). Their integrated application overcomes the limitations of single-modal detection in diagnostic accuracy and prognostic predictability and realizes the transformation of CHD diagnosis from anatomical stenosis evaluation to pathological and functional comprehensive assessment.

A standardized stepwise algorithm for the integrated application of imaging and biomarkers in CHD diagnosis and prognosis has been formed based on clinical evidence and guideline recommendations, with the specific implementation steps as follows: first, conduct preliminary population risk stratification by evaluating the baseline risk of CHD in subjects using traditional risk factors (age, gender, hypertension, diabetes, dyslipidemia, smoking history) combined with PRS and basic metabolic indicators (blood lipid, blood glucose); second, perform circulating biomarker screening by detecting core biomarkers in high-risk and medium-risk populations, including inflammatory biomarkers (hs-CRP, IL-1β), plaque instability biomarkers (MMPs, PAPP-A), and metabolic biomarkers (TMAO, adiponectin), to further identify individuals with elevated pathological risk; third, carry out anatomical imaging evaluation by performing CCTA on biomarker-positive populations to assess coronary artery stenosis degree, plaque burden, and plaque morphological characteristics (calcified, non-calcified, mixed plaque) and exclude subjects with no obvious anatomical lesions; fourth, conduct metabolic and functional imaging detection by using ^18^F-FDG PET or stress perfusion CMR for populations with non-obstructive stenosis or high-risk morphological plaques to detect the inflammatory metabolic activity of plaques and myocardial perfusion function, and identify vulnerable plaques and myocardial ischemic areas; finally, implement comprehensive risk stratification and clinical decision-making by classifying the subjects into low, medium, and high risk according to the combined results of imaging and biomarkers, where low-risk subjects receive lifestyle intervention and regular follow-up. Medium-risk subjects are given optimized medical therapy, and high-risk subjects with vulnerable plaques or myocardial ischemia receive intensive drug treatment and close clinical monitoring, with revascularization intervention performed when necessary (237).

This integrated algorithm realizes the multidimensional characterization of CHD from systemic risk to local lesion and from anatomical structure to pathological function, which can significantly improve the early identification rate of high-risk individuals, accurately predict the occurrence of MACE, and provide a scientific basis for personalized and precise management of CHD patients. In clinical practice, the algorithm can be flexibly adjusted according to the clinical characteristics of patients and the technical conditions of medical institutions, and its clinical applicability and prognostic value have been verified in multiple prospective cohort studies (39).

### 5.4 Therapeutic windows and time-dependent intervention strategies

Despite the promising therapeutic potential of targeting endothelial dysfunction and epigenetic regulation in atherosclerosis and related vascular diseases, the optimal therapeutic window for intervention remains largely underappreciated. Vascular injury and atherosclerotic progression evolve through distinct phases, each characterized by unique pathological mechanisms that directly influence treatment efficacy (238).

In the early or acute phase of vascular injury, which corresponds to initial endothelial dysfunction, mild inflammation, and reversible barrier impairment, interventions aimed at restoring endothelial homeostasis, suppressing acute pro-inflammatory signaling, and reversing aberrant epigenetic modifications are particularly effective (239). During this stage, pathological changes remain plastic, and timely treatment can prevent lipid accumulation, inflammatory cell infiltration, and EndMT, thereby halting lesion initiation.

In the subacute phase, characterized by sustained inflammation, vascular smooth muscle cell proliferation, and extracellular matrix deposition, therapeutic strategies should focus on inhibiting inflammatory amplification, blocking harmful epigenetic crosstalk, and stabilizing nascent atherosclerotic lesions (240). Delayed intervention at this stage can still attenuate plaque progression but requires combined targeting of both cellular and epigenetic mechanisms.

In the chronic phase, advanced plaques with established fibrosis, necrotic core formation, and impaired vascular remodeling represent a less reversible stage (241). At this point, interventions are less effective at reversing lesions and are instead directed toward plaque stabilization, reducing thrombotic risk, and preventing acute cardiovascular events.

Therefore, time-dependent, phase-specific therapeutic strategies are essential to maximize efficacy. Future investigations should focus on identifying precise molecular signatures that define distinct phases of endothelial dysfunction and atherosclerosis, enabling personalized and temporally optimized interventions. Developing epigenetic and endothelial-targeted therapies with well-defined therapeutic windows will greatly improve clinical translation and outcome in vascular disease management.

## 6 Treatment strategies and new directions of intervention

The treatment of CHD is entering a new stage of multilevel and precise intervention. Traditional treatments focus on relieving stenosis and preventing thrombosis. New strategies aim to regulate the metabolism-inflammation-immunity network, stabilize vulnerable plaques, and repair vascular function.

Current treatment combines lifestyle management, drug therapy, and revascularization (242). It also explores new methods such as cell therapy and targeted molecular intervention. The goal is to improve long-term prognosis and reduce the risk of acute events.

### 6.1 Optimization of traditional treatment strategies

Traditional treatments are the foundation of CHD management. Recent progress has focused on optimizing their use to make them more effective and personalized.

#### 6.1.1 Lifestyle intervention and primary prevention

Lifestyle intervention is the first line of defense for both prevention and treatment (243). It includes a healthy diet, regular exercise, smoking cessation, and limiting alcohol intake.

A heart-healthy diet emphasizes low fat, low salt, and high fiber. It reduces saturated fat and cholesterol to control LDL levels. Regular moderate exercise, such as brisk walking, can improve vascular elasticity, reduce inflammation, and enhance insulin sensitivity (244).

Smoking cessation is the most effective single measure to reduce CHD risk. It can quickly improve endothelial function and lower the chance of plaque rupture. For high-risk groups, such as those with a family history or high PRS, early lifestyle intervention can delay or even prevent the onset of the disease (245).

#### 6.1.2 Progress in drug therapy

Drug therapy aims to control symptoms, stabilize plaques, and prevent cardiovascular events. Key drug classes have seen important updates in recent years.

Lipid-lowering drugs remain the core treatment (246). Statins are still the first choice. They lower LDL and have anti-inflammatory effects. Newer drugs, such as PCSK9 inhibitors, can further reduce LDL levels in patients who do not respond well to statins. Inclisiran, a small interfering RNA drug, can lower LDL for a long time with just a few injections per year (247).

Antiplatelet drugs prevent thrombosis. Aspirin is the basic drug. Newer P2Y12 inhibitors, like ticagrelor, act faster and have a lower risk of bleeding in some cases. For patients with acute coronary syndrome, a longer period of dual antiplatelet therapy may be needed, but the duration is now decided based on individual risk (248).

Antihypertensive and antidiabetic drugs also play a key role (249). Renin-angiotensin system inhibitors can protect the endothelium and reduce vascular remodeling. SGLT2 (sodium-glucose cotransporter 2) inhibitors and GLP-1 (glucagon-like peptide-1) agonists, originally used for diabetes, have been shown to reduce heart failure and cardiovascular death in CHD patients, even those without diabetes.

#### 6.1.3 Permission to reuse and copyright

Revascularization quickly restores blood flow to the heart muscle (250). It includes PCI (percutaneous coronary intervention) and CABG (coronary artery bypass grafting).

PCI, especially with drug-eluting stents, is widely used for acute coronary syndrome and stable CHD. Newer bioresorbable scaffolds can be absorbed by the body over time, leaving a normal blood vessel (251). Intravascular imaging (IVUS/OCT) is now commonly used during PCI to ensure optimal stent placement.

CABG is more suitable for patients with severe multi-vessel disease or left main coronary artery disease. It provides better long-term results in these high-risk groups. Minimally invasive CABG reduces surgical trauma and shortens recovery time (252).

The choice between PCI and CABG is now based on a comprehensive assessment of the coronary anatomy, myocardial function, and the patient’s overall condition (253).

### 6.2 New directions of targeted intervention based on pathological mechanisms

New treatments target the core pathological processes of CHD, such as inflammation, immune disorder, and metabolic imbalance. These methods aim to treat the root cause rather than just the symptoms.

#### 6.2.1 Anti-inflammatory therapy and immune regulation

Since inflammation is a key driver of atherosclerosis, anti-inflammatory therapy has become a hot research topic (254).

Canakinumab, a monoclonal antibody against IL-1β, has been shown to reduce cardiovascular events in patients with high inflammation levels, even when LDL is well-controlled. Colchicine, an old drug for gout, can also lower the risk of recurrent events in CHD patients by inhibiting inflammation (255).

Immune regulation focuses on restoring the balance of immune cells. Targeting the CD40-CD40L pathway or inhibiting excessive macrophage activation are potential strategies. Treg therapy is also being studied to suppress harmful immune responses in the blood vessels (256).

#### 6.2.2 Metabolic regulation and improvement of endothelial function

Correcting metabolic disorders is crucial for long-term CHD management. Apart from controlling blood sugar and lipids, new therapies target cellular metabolism (257). For example, drugs that improve mitochondrial function can reduce oxidative stress and protect the endothelium. Modulating fatty acid oxidation in the heart can also improve myocardial energy supply (258).

Endothelial repair therapy uses drugs to promote the production of nitric oxide (NO) and reduce endothelial apoptosis (259). Stem cell-derived exosomes are being tested to repair damaged endothelium and promote vascular regeneration (260).

#### 6.2.3 Targeted intervention for vulnerable plaques

Identifying and stabilizing vulnerable plaques is key to preventing acute myocardial infarction (261).

Drug-eluting balloons can deliver antiproliferative drugs to the plaque site without leaving a permanent stent (262). This is useful for treating small vessels or in-stent restenosis.

Local drug delivery systems, such as nanoparticles, can carry anti-inflammatory or lipid-lowering drugs directly to the vulnerable plaque (263). This reduces systemic side effects and increases the drug concentration at the target site.

Gene therapy is another approach (264). It aims to increase the expression of genes that strengthen the fibrous cap or reduce inflammation in the plaque.

### 6.3 Emerging treatment technologies and future prospects

Several cutting-edge technologies are under development and hold promise for the future of CHD treatment (265).

#### 6.3.1 Cell therapy and regenerative medicine

Cell therapy aims to replace damaged myocardial cells and repair heart function (266).

MSCs (mesenchymal stem cells) are the most studied (267). They can reduce inflammation, promote angiogenesis, and improve cardiac function after a heart attack. Cardiac progenitor cells are being tested to directly regenerate heart muscle (268).

Although early results are promising, challenges remain (269). For example, ensuring the cells survive and function properly in the heart is a key issue.

#### 6.3.2 Precision medicine and individualized treatment

Precision medicine uses a patient’s genetic information, biomarkers, and lifestyle to design a personalized treatment plan (270).

PRS can help identify patients who need more aggressive lipid-lowering therapy (271). Biomarkers like hs-CRP or TMAO can guide the use of anti-inflammatory or metabolic drugs (272).

Pharmacogenomics studies how genes affect a patient’s response to drugs (273). This can help avoid adverse reactions and choose the most effective medication.

#### 6.3.3 Multilevel combined intervention strategy

The most effective future treatment will likely be a combination of different strategies (274). For example, a patient with high genetic risk may receive early statin therapy plus lifestyle intervention. A patient with acute coronary syndrome may undergo PCI, followed by antiplatelet drugs and colchicine for inflammation. A patient with metabolic disorders may use SGLT2 inhibitors together with dietary guidance.

This multilevel approach addresses the complex nature of CHD (275). It combines local treatment of the blood vessels with systemic regulation of the body’s networks.

In conclusion, the treatment of CHD is evolving rapidly. Traditional therapies are being refined, whereas new targeted treatments are emerging. The future will focus on early intervention, precise targeting of pathological mechanisms, and personalized care based on each patient’s unique characteristics. This comprehensive strategy will greatly improve the prevention and treatment of CHD.

## 7 Summary and outlook

CHD is a complex chronic disease (276). Its occurrence and development depend on the interaction of multiple factors, rather than a single pathological change. In recent years, research has found that CHD is not only a local vascular disease caused by atherosclerosis but also a systemic disorder involving metabolism, inflammation, and immunity (277).

This review summarizes the latest progress in CHD research, and we now understand that endothelial dysfunction is the starting link of the disease (278). Abnormal lipid metabolism provides the material basis for plaque formation, and immune-inflammatory responses run through the whole process of plaque formation, maturation, and rupture. The MII network is the core mechanism to connect these factors, and it explains why metabolic diseases and chronic inflammation significantly increase the risk of CHD (225).

In clinical practice, the understanding of CHD has also changed greatly these days (279). We now know that the degree of coronary stenosis is not the only factor to determine the severity of CHD. Plaque stability, microvascular function, and systemic metabolic state are equally important in the process, and the change has promoted the transformation of diagnostic technology (280). Diagnosis of CHD is now no longer limited to finding stenosis, and it focuses on identifying vulnerable plaques, assessing myocardial perfusion, and detecting early molecular changes.

New biomarkers and imaging technologies have now improved the accuracy of early diagnosis and risk stratification (281). Combining traditional risk factors with PRS and molecular markers, has made risk assessment more personalized, and this allows doctors to identify high-risk groups earlier and take targeted preventive measures.

In the field of treatment, the strategy has developed from treating the lesion to multilevel intervention (282). Traditional treatments such as lipid-lowering drugs, antiplatelet therapy, and revascularization are still the foundation. However, their use is becoming more refined and personalized. At the same time, new treatment directions are emerging. Anti-inflammatory therapy, metabolic regulation, and targeted intervention on vulnerable plaques have shown good prospects. These new methods aim to treat the root causes of the disease and improve long-term prognosis.

Despite these advances, many challenges remain (283). First, the translation of basic research results into clinical practice is still slow. Many promising targets in animal experiments have not yet been successfully applied to human treatment. Second, the early intervention of CHD needs to be strengthened (284). We still lack simple and effective methods to detect pre-plaque lesions in large populations. Third, the complexity of the disease makes it difficult to develop single-target drugs. Most patients need combined treatment, but the optimal combination scheme for different individuals is not yet clear.

Looking to the future, the prevention and treatment of CHD will focus on three directions. First is precision medicine (285). With the development of genomics and metabolomics, we will be able to design treatment plans according to each patient’s genetic background, metabolic characteristics, and immune state. This will avoid the one-size-fits-all treatment model and improve the therapeutic effect.

Second is early intervention and primary prevention (286). We need to shift the focus of treatment from the late stage of the disease to the early stage and even the pre-disease stage. By intervening in the metabolism-inflammation-immunity network early, we can delay or even reverse the occurrence of atherosclerosis. Lifestyle management will remain the most economical and effective measure for primary prevention.

Third is the development of multitarget combined therapy (287). Since CHD is driven by a complex network, single-target drugs are often difficult to achieve ideal results. The future will see more combined therapies that target multiple links of the disease at the same time—for example, combining lipid-lowering drugs with anti-inflammatory drugs, or combining revascularization with cell therapy for myocardial repair.

In addition, emerging technologies such as artificial intelligence and big data will also play an important role (288). They can help analyze complex clinical and biological data, optimize risk assessment models, and assist doctors in making more accurate diagnosis and treatment decisions. The effectiveness hierarchy and limitations of different treatment strategies for CHD are shown in **Table 4**.

**Table 4:** The effectiveness hierarchy and limitations of different treatment strategies for CHD.

Treatment strategy	Main target	Intervention level	Main clinical benefit	Level of evidence	Limitations	References
Statins	LDL	Metabolism	Reduce MACE, lower LDL-C levels, inhibit plaque inflammation	Guidelines/RCT	Limited control over inflammation, individual lipid-lowering response variability	Mach F, 2020 (Eur Heart J) (191); Ridker PM, 2008 (N Engl J Med) (194)
Antiplatelet therapy	Thrombosis	Blood	Prevent intra-plaque thrombosis, reduce ACS incidence, lower post-PCI ischemic events	Guidelines/RCT	Does not reverse plaque, increased bleeding risk in long-term use	Valgimigli M, 2018 (Eur Heart J) (202); Palmerini T, 2015 (J Am Coll Cardiol) (289)
Revascularization	Local stenosis	Organ	Rapid relief of myocardial ischemia symptoms, improve cardiac function, reduce refractory angina mortality	Guidelines/RCT	Systemic risk remains, invasive procedure-related complications, in-stent restenosis risk	Knuuti J, 2020 (Eur Heart J) (56); Maron DJ, 2020 (N Engl J Med) (228)
Anti-inflammatory therapy	IL-1β	Inflammation	Reduce recurrent cardiovascular events in high-inflammatory patients, stabilize vulnerable plaques, lower MACE risk in statin-treated patients	RCT/clinical trial	Significant individual variability, increased serious infection risk, no benefit in low-inflammation populations	Ridker PM, 2017 (N Engl J Med) (152); Tardif JC, 2019 (N Engl J Med) (122)

ACS, acute coronary syndrome; IL-1β, interleukin-1β; LDL, low-density lipoprotein; LDL-C, low-density lipoprotein cholesterol; MACE, major adverse cardiovascular events; PCI, percutaneous coronary intervention; RCT, randomized controlled trial.

In conclusion, research on CHD has made remarkable progress in recent years. Our understanding of its pathological mechanisms has become deeper, and diagnostic and treatment methods have become more diverse and precise. Although there are still many problems to be solved, with the continuous development of medical science and technology, we are moving toward a new era of precise prevention and treatment of CHD. The goal of reducing the incidence and mortality of this disease is expected to be better achieved in the future.
